# Design and Evaluation of a Multi-Epitope Vaccine Targeting Conserved Envelope and NS5 Proteins of Usutu Virus Using Immunoinformatics

**DOI:** 10.3390/microorganisms14092026

**Published:** 2026-09-11

**Authors:** Reem Alromaihi, Hajed Obaid Alharbi, Suleman Abdullah Almerdasi, Mawahib A. Ahmed, Waad A. Aljohani, Mona Alromaihi, Laila Alhussain, Alaa Karkashan, Riham Mohamad Rashad Mohamad, Khaled S. Allemailem

**Affiliations:** 1 Department of Medical Laboratories, College of Applied Medical Sciences, Qassim University, Buraydah 51452, Saudi Arabias.almerdasi@qu.edu.sa (S.A.A.);; 2 Department of Basic Health Sciences, College of Applied Medical Sciences, Qassim University, Buraydah 51452, Saudi Arabia; 3 Department of Pediatrics, College of Medicine, Qassim University, Buraydah 51452, Saudi Arabia; mona.alromaihi@qu.edu.sa; 4 Department of Biology, College of Science, Qassim University, Buraydah 51452, Saudi Arabia; 5 Department of Biological Sciences, College of Science, University of Jeddah, Jeddah 21959, Saudi Arabia; 6 Department of Laboratory and Blood Bank, Buraydah Central Hospital, Buraydah 52361, Saudi Arabia

**Keywords:** Usutu virus, reverse vaccinology, Envelope protein, NS5 protein, molecular docking, molecular dynamics

## Abstract

Usutu virus is an emerging mosquito-borne flavivirus with an expanding geographic distribution and increasing public health relevance, yet no licensed vaccine is currently available. This study used an integrated reverse vaccinology strategy to identify conserved immunogenic regions from the Envelope protein and NS5 protein, and construct a multi-epitope vaccine. Following sequential computational screening, the retained T-cell and B-cell epitopes satisfied the predefined selection criteria, while selected T-cell epitopes achieved an estimated 96.41% global population coverage. The final vaccine consisted of 240 amino acids and incorporated an adjuvant together with peptide linkers. Computational characterization indicated favorable physicochemical features and a refined three-dimensional model with improved stereochemical characteristics. Receptor-binding analyses predicted favorable interactions with TLR2 and TLR4, producing weighted docking scores of −1326.1 and −1230.2, respectively. Molecular dynamics simulation further characterized the temporal behavior of the vaccine–TLR2 complex, while MM-GBSA analysis yielded an estimated binding energy of −74.78 kcal/mol. C-ImmSim predicted enhanced humoral and cellular immune-response patterns following repeated antigen administration, including increased simulated antibody levels and changes in immune-cell populations. All findings in this study are based on in silico analyses and represent computational predictions rather than experimentally confirmed results. Further experimental validation is required to verify the predicted properties, immunogenicity, and protective potential of the proposed vaccine candidate.

## 1. Introduction

Usutu virus (USUV) is an emerging mosquito-borne virus belonging to the *Flaviviridae* family and the *Flavivirus* genus [1]. The virus was first isolated in 1959 in southern Africa and was named after the Usutu River in Eswatini [2]. In nature, Usutu virus is maintained primarily through a transmission cycle involving mosquitoes and birds [3]. Mosquitoes, particularly those of the *Culex* genus, act as the main vectors of USUV, while birds serve as natural reservoir hosts [4]. Due to its zoonotic potential and its impact on wildlife and human health, Usutu virus has attracted increasing attention in recent years. Despite its public health significance, no specific licensed vaccine or antiviral treatment is currently available for USUV infection [1]. As a result, treatment of USUV infection is largely supportive, as no specific antiviral treatment is currently available [5].

Vaccination continues to be an important and widely used approach for the prevention of viral infections and associated diseases [6]. In contrast to antibiotics, which are used to treat bacterial infections, vaccines stimulate the immune system to recognize pathogen-derived antigens and generate immune responses that can contribute to protection against viral infections [7]. Progress in computational biology and immunoinformatics has contributed substantially to the development of new vaccine design strategies [8,9]. Notably, multi-epitope vaccine formulations represent a promising vaccination approach because they combine several antigenic determinants within a single construct, potentially allowing the induction of both humoral and cell-mediated immune responses [10]. Reverse vaccinology uses genome and proteome-derived information to identify suitable vaccine targets and support the computational identification of immunogenic epitopes [11]. Such in silico approaches can reduce the time and cost associated with vaccine development while supporting systematic multi-epitope vaccine design and the computational assessment of immunological and safety-related properties [12].

Previous computational investigations of Usutu virus have primarily focused on the identification and characterization of potential immunogenic epitopes. Computational analysis of the Envelope protein has identified candidate B-cell and T-cell epitopes with predicted HLA-binding capacity and population coverage [13], while subsequent investigations of multiple non-structural proteins have reported numerous continuous and discontinuous B-cell epitopes as well as MHC class I- and class II-binding peptides [14]. These studies have provided valuable insights into the potential antigenic regions of Usutu virus. However, they lacked comprehensive structural and molecular characterization of an integrated multi-epitope vaccine.

In the present investigation, the Envelope protein and NS5 protein were selected based on their biological relevance, sequence conservation, predicted antigenicity, and absence of predicted allergenicity. The Envelope protein was selected because of its surface-associated role in host–cell attachment and membrane fusion, whereas the NS5 protein represents an essential non-structural protein involved in viral genome replication, providing antigenic targets associated with distinct stages of the viral life cycle. The selected B-cell epitopes, cytotoxic T-lymphocyte epitopes, and helper T-lymphocyte epitopes were identified using the IEDB Next Generation Epitope Prediction Pipeline and integrated into a single multi-epitope vaccine. The construct was subsequently subjected to physicochemical analysis, three-dimensional structure prediction and refinement, discontinuous B-cell epitopes mapping, molecular docking with TLR2 and TLR4, molecular dynamics simulation, MM-GBSA analysis, immune-response simulation, and in silico cloning. Thus, the present study extends previous epitope-focused investigations of Usutu virus by integrating conserved Envelope protein- and NS5 protein-derived epitopes into a unified multi-epitope vaccine and evaluating its predicted structural, molecular, and immunological characteristics through a comprehensive immunoinformatics approach.

## 2. Materials and Methods

**Figure 1:**
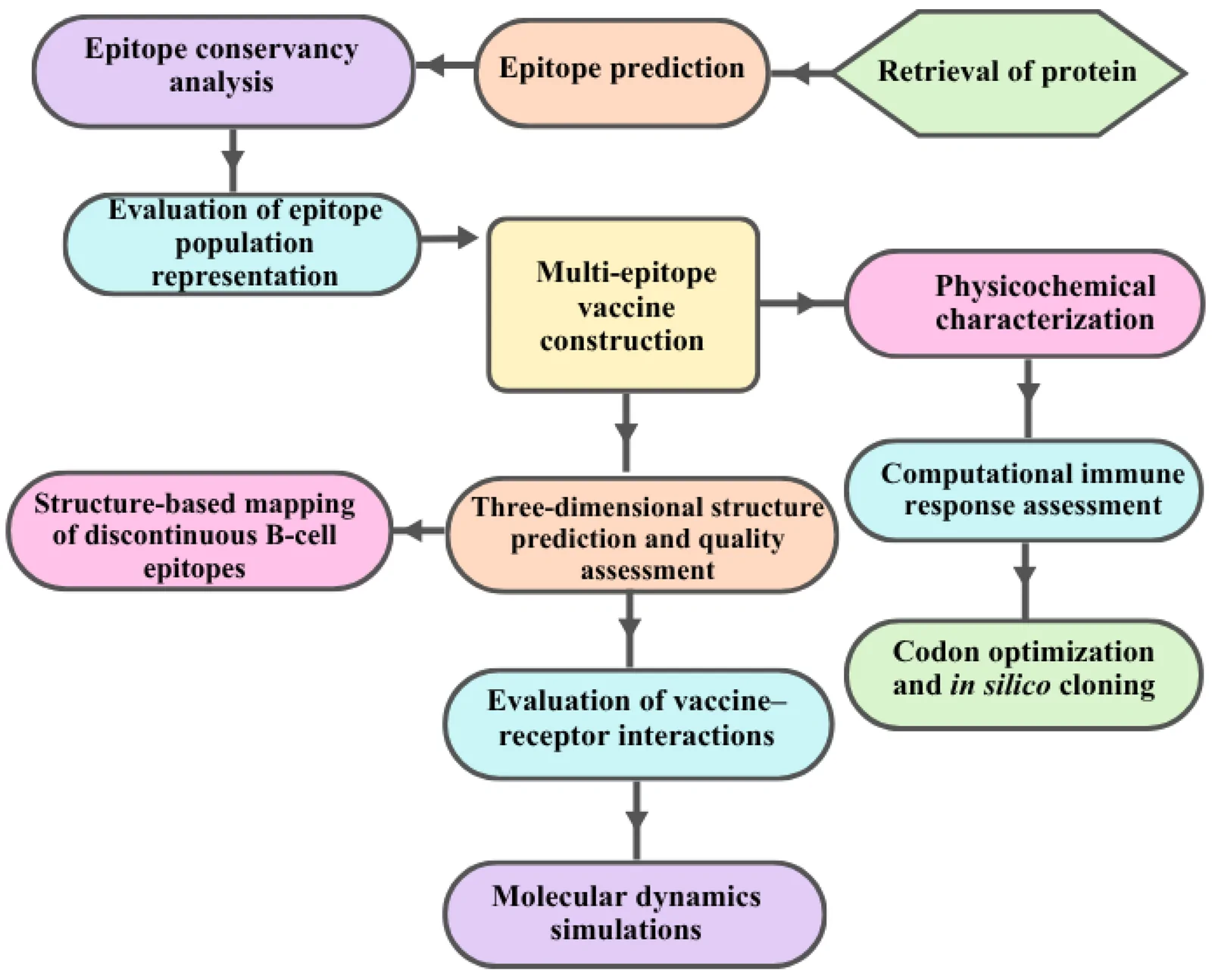
Schematic overview of the computational strategy used for the design and evaluation of the multi-epitope vaccine against Usutu virus.

Figure 1 summarizes the computational strategy followed in this study. The workflow progresses from protein retrieval and epitope identification to construction of the multi-epitope vaccine, followed by comprehensive computational evaluation to assess its predicted suitability.

### 2.1. Retrieval of Protein

The Envelope protein and non-structural protein 5 (NS5) encoded by the Usutu virus polyprotein were selected for the present investigation. Their amino acid sequences were downloaded from the UniProt database (https://www.uniprot.org/, accessed on 12 July 2026) [15] using accession numbers A0A7S5IEW7 (Envelope protein) and A0A075W8L0 (NS5 protein). To examine sequence conservation, homologous proteins were identified by performing BLASTp (https://blast.ncbi.nlm.nih.gov/Blast.cgi?PROGRAM=blastp, accessed on 12 July 2026) searches against the NCBI non-redundant protein database. From the retrieved homologs, 200 sequences meeting the selection criteria of >98% query coverage and >98% sequence identity were retained for subsequent analyses. This stringent threshold was applied to retain highly similar and sufficiently complete homologous sequences while minimizing the inclusion of distantly related sequences and reducing potential alignment inconsistencies during residue-level conservation analysis. The selected sequences represented closely related USUV homologs used for conservation analysis. The complete sets of 200 homologous sequences corresponding to the Envelope protein and NS5 protein derived from the Usutu virus polyprotein are provided as Supplementary Material S1 to facilitate reproducibility. Multiple sequence alignment was carried out in BioEdit v7.7.1.0 software using the ClustalW algorithm. Conservation at individual amino acid positions was quantified using Shannon entropy (Hx) values generated within BioEdit software. Residues with entropy values below 0.5 were interpreted as conserved, whereas higher values indicated increased sequence variation. The resulting conservation profile was subsequently used to identify conserved regions suitable for downstream vaccine design. TMHMM Server v2.0 (https://services.healthtech.dtu.dk/services/TMHMM-2.0/, accessed on 12 July 2026) was used to screen the selected proteins for transmembrane helices [16]. Antigenicity was subsequently examined using VaxiJen v3.0 (https://www.ddg-pharmfac.net/vaxijen3/home/, accessed on 12 July 2026) [17], retaining only proteins that satisfied the recommended score criterion. Protein allergenicity was then assessed using AllerTOP v2.1 (https://www.ddg-pharmfac.net/allertop_v2/, accessed on 12 July 2026) [18].

### 2.2. Epitope Prediction

The antigenic proteins were used as the source for epitope prediction. ABCPred server (https://webs.iiitd.edu.in/raghava/abcpred/ABC_submission.html, accessed on 14 July 2026) was used to predict linear B-cell epitopes using a threshold of 0.51 [19]. The IEDB Next-Generation Epitope Prediction Pipeline was used to identify CTL-binding peptides through NetMHCpan 4.1 EL (https://nextgen-tools.iedb.org/pipeline?tool=tc1, accessed on 14 July 2026) [20], whereas NetMHCIIpan 4.1 EL (https://nextgen-tools.iedb.org/pipeline?tool=tc2, accessed on 14 July 2026) was used to predict the corresponding HTL peptides [21]. The predicted CTL and HTL epitopes were sorted on the basis of their percentile ranks, with a percentile rank threshold of less than 1 applied to CTL epitopes, whereas HTL epitopes with percentile rank scores of less than 10 were selected. For CTL epitope prediction, the Human: 27 Allele Panel was used. For HTL epitope prediction, 11 HLA-DRB1 alleles, namely HLA-DRB1*01:01, HLA-DRB1*03:01, HLA-DRB1*04:01, HLA-DRB1*04:05, HLA-DRB1*07:01, HLA-DRB1*08:02, HLA-DRB1*09:01, HLA-DRB1*11:01, HLA-DRB1*12:01, HLA-DRB1*13:02, and HLA-DRB1*15:01, were selected to provide broad HLA representation and maximize potential population coverage. Antigenicity was predicted using VaxiJen v3.0. The tool classified antigenic epitopes as “Probable immunogen” with a probability of either 66% or 100%, and these epitopes were retained for further analysis. For non-antigenic epitopes, the tool provided the classification “Probable non-immunogen” with a probability of either 66% or 100%, and these epitopes were excluded from further analysis. Allergenicity and toxicity were subsequently assessed using AllerTOP v2.1 and ToxinPred v1.0 (https://webs.iiitd.edu.in/raghava/toxinpred/, accessed on 12 July 2026) [22], respectively, using the default prediction criteria of the respective tools. Sequence cross-reactivity analysis was subsequently performed using BLASTp against *Homo sapiens* reference proteins (taxid: 9606), using an E-value threshold of ≤0.001 to identify significant sequence similarities between the selected vaccine epitopes and human proteins. Only epitopes that satisfied all the required filtering criteria were considered for assembly of the multi-epitope vaccine.

### 2.3. Epitope Conservancy Analysis

The conservancy of the selected B-cell, CTL, and HTL epitopes was evaluated using the IEDB Epitope Conservancy Analysis tool (https://tools.iedb.org/conservancy/, accessed on 18 July 2026) [23]. The same sets of 200 homologous protein sequences used for the Shannon entropy analysis were used for epitope conservancy assessment, with the corresponding 200 sequences analyzed separately for the Envelope protein and NS5 protein. The selected epitope sequences were evaluated against their respective homologous protein sequence sets using the default parameters of the tool.

### 2.4. Evaluation of Epitope Population Representation

The IEDB Population Coverage Tool (https://tools.iedb.org/population/, accessed on 19 July 2026) was used to estimate the predicted population representation of the selected T-cell epitopes [24]. Combined population coverage analysis was performed using both CTL and HTL epitopes, incorporating the associated HLA alleles across the world population. The analysis was performed using the default parameters of the tool. This analysis estimates the proportion of individuals predicted to present one or more of the selected CTL and HTL epitopes based on the corresponding HLA alleles. This computational estimate represents predicted HLA population coverage and does not indicate vaccine efficacy, clinical protection, or experimentally demonstrated immune responses.

### 2.5. Multi-Epitope Vaccine Construction

Construction of the multi-epitope vaccine involved the sequential integration of the selected B-cell, CTL, and HTL epitopes into a single polypeptide. KK linkers were used to connect B-cell epitopes, AAY linkers were used to connect CTL epitopes, and GPGPG linkers were introduced between HTL epitopes to maintain separation between the epitope sequences. To enhance the immunomodulatory properties of the multi-epitope vaccine construct, human β-defensin-3 was incorporated at the N-terminal end as an adjuvant, while the PADRE sequence was incorporated to enhance helper T-cell responses. A 6×His tag was positioned at the C-terminal end to support purification following recombinant expression. Human β-defensin-3 was selected based on its reported immunomodulatory properties and its ability to interact with pathways involved in both innate and adaptive immune responses, supporting its potential application as an endogenous vaccine adjuvant [25]. Previous studies have reported that human β-defensin-3 can function as an endogenous adjuvant by promoting the maturation and activation of antigen-presenting cells and activating professional antigen-presenting cells through Toll-like receptor 1/2 (TLR1/TLR2)-dependent signaling, thereby supporting antigen-specific immune responses [26]. Human β-defensin-3 has also been reported to modulate NF-κB signaling and cytokine production through the TLR1/TLR2 pathway in human monocytes, further supporting its potential role in innate immune modulation and vaccine adjuvant design [27]. Furthermore, human β-defensin-3 has been reported to regulate immune responses through the LPS/TLR4-mediated signaling pathway [28]. The β-defensin-3 adjuvant was connected to the first epitope using an EAAAK linker. The completed construct was subsequently assessed for antigenicity and allergenicity using VaxiJen v3.0 and AllerTOP v2.1, respectively.

### 2.6. Physicochemical Characterization

The ExPASy ProtParam server (https://web.expasy.org/protparam/, accessed on 21 July 2026) was used to characterize the physicochemical profile of the multi-epitope vaccine [29]. The predicted physicochemical parameters were used to examine the overall protein characteristics and support downstream computational analyses. Protein solubility under recombinant *Escherichia coli* expression conditions was estimated with SoluProt v1.0 (https://loschmidt.chemi.muni.cz/soluprot/, accessed on 21 July 2026) [30]. A SoluProt score of 0.5 was used as the threshold, with scores above 0.5 considered indicative of predicted solubility. The resulting prediction provided an initial indication of the construct’s suitability for heterologous expression.

### 2.7. Three-Dimensional Structure Prediction and Quality Assessment

The three-dimensional model of the designed multi-epitope vaccine was predicted by using AlphaFold3 server (https://alphafoldserver.com/, accessed on 21 July 2026) [31]. The AlphaFold3 prediction was further evaluated using the predicted template modeling (pTM) score and predicted aligned error (PAE) plot to assess the confidence of the predicted structure. The predicted structure subsequently underwent refinement using GalaxyRefine (https://galaxy.seoklab.org/cgi-bin/submit.cgi?type=REFINE, accessed on 21 July 2026) to improve overall model geometry and optimize side-chain conformations [32]. Following refinement, stereochemical quality was assessed with PROCHECK through the UCLA-DOE LAB SAVES v6.1 platform (https://saves.mbi.ucla.edu/, accessed on 21 July 2026), and Ramachandran plot statistics were examined to determine residue distribution among favored, additionally allowed, generously allowed, and disallowed regions. Structural quality was further assessed using ERRAT, providing an independent evaluation of the refined protein model prior to subsequent computational analyses [33].

### 2.8. Structure-Based Mapping of Discontinuous B-Cell Epitopes

To predict conformational B-cell epitopes from the refined three-dimensional vaccine model, the ElliPro server (https://tools.iedb.org/ellipro/, accessed on 22 July 2026) available through the IEDB, was used [34]. The refined vaccine structure served as the input for this analysis. The method identifies potential antibody-interacting regions by evaluating residue spatial distribution and grouping exposed amino acids into surface clusters likely to participate in antibody binding. Default settings were used during the analysis, where epitope clusters were defined by applying a protrusion index cutoff value of 0.5 along with a residue separation limit of 6 Å.

### 2.9. Evaluation of Vaccine–Receptor Interactions

Interactions between the designed multi-epitope vaccine and the innate immune receptors TLR2 (PDB ID: 6NIG) and TLR4 (PDB ID: 3FXI) were investigated through molecular docking. TLR2 is primarily involved in the recognition of bacterial lipoproteins and lipopeptides and plays an important role in the activation of antigen-presenting cells and the induction of pro-inflammatory cytokines [35,36]. In comparison, TLR4 is a key component of innate immune signaling and contributes to the production of cytokines and costimulatory molecules, thereby supporting downstream immune pathways involved in adaptive immunity [37]. The complete receptor structures were used for the docking analysis. Prior to docking, the receptor structures were prepared using Discovery Studio v25.1.0, where crystallographic water molecules and attached ligands were removed using the default receptor-preparation parameters. The refined vaccine model served as the docking ligand, whereas ClusPro 2.0 (https://cluspro.bu.edu/login.php, accessed on 22 July 2026) was used for molecular docking [38]. The platform generated multiple binding conformations, which were prioritized according to weighted docking scores along with cluster size distribution. Complexes exhibiting favorable weighted docking scores together with substantial cluster populations were retained for detailed investigation. Interaction profiling of the selected vaccine–receptor complexes was completed with PDBsum server (https://www.ebi.ac.uk/thornton-srv/databases/pdbsum/Generate.html, accessed on 23 July 2026) [39]. For interaction analysis, the receptor chain exhibiting the best binding score was considered.For TLR2, the interactions were reported for chain D. The selected TLR2–vaccine complex was further analyzed using COCOMAPS 2.0 (https://aocdweb.com/BioTools/cocomaps2/, accessed on 26 August 2026) to characterize the interface and residue-level atomic interaction patterns [40]. The analysis included interface area, buried surface area, interacting residues, proximal contacts, hydrogen bonds, van der Waals contacts, salt bridges, π–π interactions, CH–π interactions, and other interface contacts. The selected TLR2–vaccine complex was subsequently subjected to molecular dynamics (MD) simulation. The analysis summarized the intermolecular contact patterns established between the multi-epitope vaccine and the receptor proteins, including hydrogen bonds, hydrophobic contacts, and ionic interactions. These interaction profiles were subsequently considered during the assessment of complex stability and binding characteristics.

### 2.10. Molecular Dynamics Simulations

The vaccine–TLR2 complex was subjected to molecular dynamics (MD) simulation to investigate its conformational behavior and structural persistence under physiological conditions. The docked complex served as the starting model for simulations performed with the Desmond engine integrated into the Schrödinger Suite (Schrödinger, Inc., New York, NY, USA) [41]. Before initiating the simulation, structural preparation was completed using the Maestro Protein Preparation Wizard (Schrödinger, Inc., New York, NY, USA), which included correction of bond assignments, addition of hydrogen atoms, optimization of the hydrogen-bonding network, and refinement of protonation states. The simulation environment was established within an orthorhombic simulation box, followed by explicit solvation using the TIP3P water model to represent the aqueous system. Following charge neutralization, the ionic environment was established with Na^+^ and Cl^−^ ions to obtain a final salt concentration of 0.15 M. Simulations were conducted with the OPLS-2005 force field under an NPT ensemble maintained at 300 K and 1 atm [42]. Long-range electrostatic interactions were treated using the particle mesh Ewald (PME) algorithm, while periodic boundary conditions were applied throughout the trajectory. The production simulation continued for 100 ns, and the resulting trajectories were examined to monitor changes in structural properties over time. Overall conformational behavior was characterized using RMSD, whereas residue-specific flexibility was determined using RMSF. Structural compactness was further assessed using the radius of gyration (Rg). Intermolecular interaction persistence was assessed through hydrogen-bond analysis. In addition, the binding energetics of the vaccine–TLR2 complex were estimated using the Prime MM-GBSA implementation within the Schrödinger Suite [43]. The MM-GBSA calculation was performed using one frame corresponding to the final frame of the 100 ns molecular dynamics trajectory.

### 2.11. Computational Immune Response Assessment

Immune response profiling for the designed multi-epitope vaccine was carried out using the C-ImmSim server (https://kraken.iac.rm.cnr.it/C-IMMSIM/index.php?page=1, accessed on 22 July 2026) [44,45]. The simulation was run for 1050 time steps, corresponding to approximately 350 days. Vaccine exposure was introduced at time steps 1, 84, and 168, each consisting of 1000 antigen units. The adjuvant coefficient remained 100, while LPS was excluded from the simulation environment. A random seed of 12345 and a simulation volume of 10 were used. The antigen dose and adjuvant coefficient were maintained according to the default conditions recommended by the C-ImmSim platform. These standard conditions have been adopted in multiple previous studies; therefore, no study-specific modifications were introduced.

### 2.12. Codon Optimization and In Silico Cloning

The amino acid sequence of the multi-epitope vaccine was first converted into its corresponding nucleotide sequence using EMBOSS Backtranseq (https://www.ebi.ac.uk/jdispatcher/st/emboss_backtranseq, accessed on 25 July 2026) [46]. The resulting nucleotide sequence was then subjected to codon optimization using the NovoPro Codon Optimization Tool (https://www.novoprolabs.com/tools/codon-optimization, accessed on 25 July 2026) [47]. The codon sequence was optimized for *E. coli* K12 expression while maintaining the original amino acid sequence of the multi-epitope vaccine. The optimized nucleotide sequence was subsequently modified by adding NdeI and XhoI restriction sites at its 5′ and 3′ ends, respectively. The resulting insert was then computationally cloned into the pET-28a(+) expression vector using SnapGene v8.2.1 to assess the proposed cloning strategy and confirm the organization of the recombinant plasmid before downstream experimental procedures.

## 3. Results

### 3.1. Retrieval of Protein

The Envelope protein and NS5 protein from Usutu virus were prioritized as vaccine targets because both contribute to distinct stages of the viral life cycle. The Envelope protein mediates host–cell attachment and membrane fusion, making it an important antigenic target for epitope identification. By contrast, NS5 protein functions as the viral RNA polymerase, driving synthesis of the viral genome and supporting intracellular propagation.

**Figure 2:**
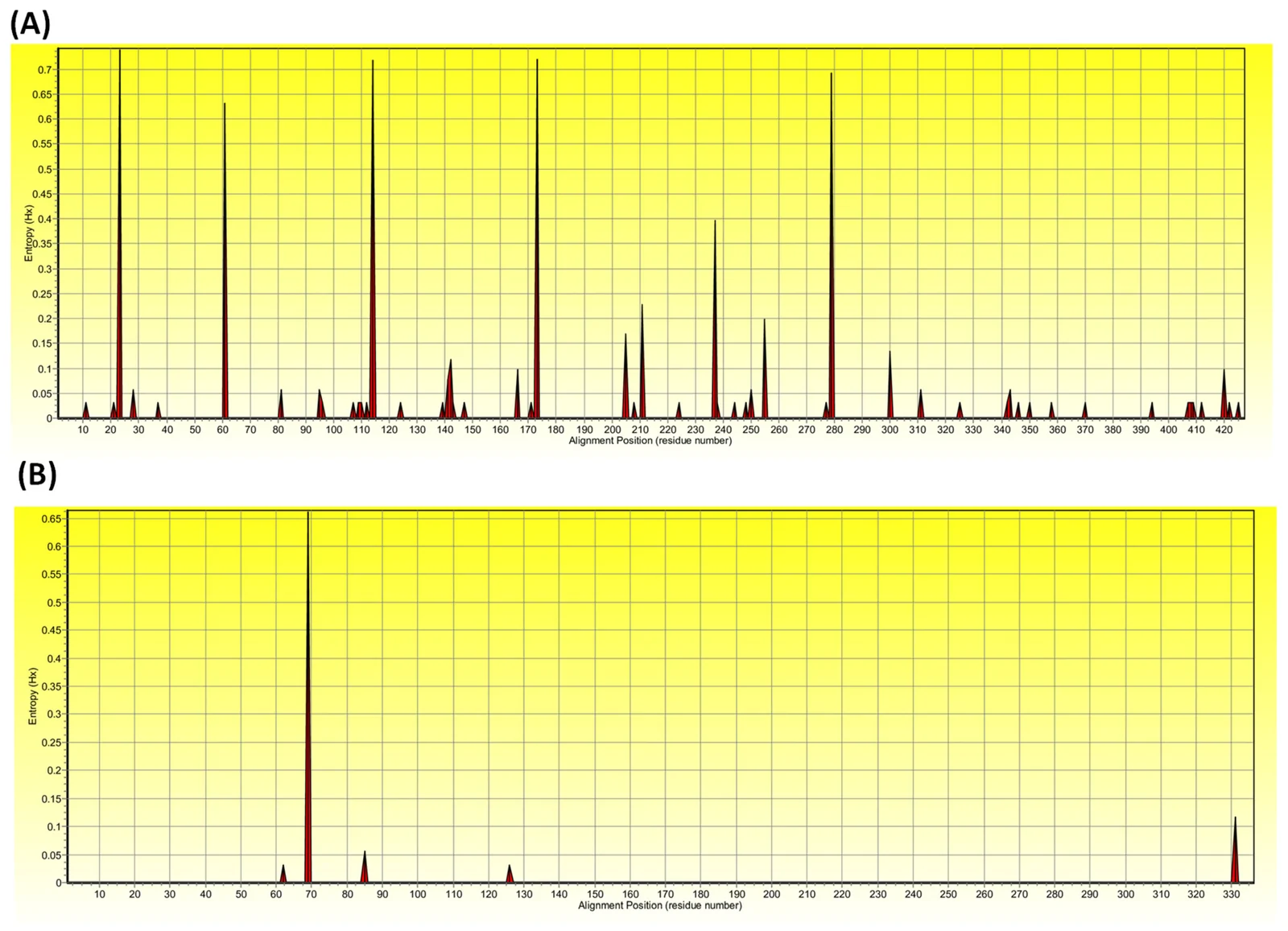
Position-specific Shannon entropy (Hx) distributions generated for the selected viral proteins based on the corresponding aligned homologous sequences. (**A**) Envelope protein. (**B**) NS5 protein. Residues with Hx values below 0.5 were considered conserved, whereas higher values indicated greater sequence variability.

Shannon entropy (Hx) measurements were used to examine conservation patterns among the aligned protein sequences. Within this analysis, lower entropy values correspond to greater sequence conservation, whereas higher values indicate increased positional variability. In the Envelope protein (Figure 2A), most amino acid positions displayed entropy values close to zero, predominantly within the 0.0–0.1 interval, indicating a high degree of conservation across most residues. However, several distinct peaks were observed at specific residue positions, where entropy values increased up to approximately 0.6–0.7, indicating localized regions of variability. Additional moderate peaks were also present in the range of 0.2–0.4, suggesting partial variability at certain positions. Despite these peaks, most residues remained highly conserved. In the case of the NS5 protein (Figure 2B), the entropy values were predominantly lower, mostly within the range of 0.0–0.05, indicating a higher degree of conservation compared to the Envelope protein. Only a few positions exhibited elevated entropy values, with a prominent peak reaching approximately 0.6, and minor peaks observed around 0.1–0.2. The limited number of variable positions indicated comparatively greater conservation of the NS5 protein. Overall, both proteins showed high conservation, with entropy values below 0.5 for the majority of residues. The Envelope protein showed relatively more variable regions compared to the NS5 protein, while the NS5 protein exhibited greater conservation across its sequence.

The antigenicity of the Envelope protein was predicted to be a probable immunogen with a probability of 66%, whereas the antigenicity of NS5 protein was predicted to be a probable immunogen with a probability of 100%. Allergenicity evaluation categorized both proteins as non-allergenic. Membrane topology analysis showed that the Envelope protein includes extracellular, intracellular, and transmembrane segments, while the NS5 protein lacked transmembrane helices. The predicted characteristics of both proteins are summarized in Table 1.

### 3.2. Epitope Prediction

Initially, a total of 29 B-cell epitopes were predicted from the Envelope protein. The NS5 protein was not used for B-cell epitope prediction. For CTL epitope prediction, 103 unique peptides were identified from the Envelope protein and 93 unique peptides were identified from the NS5 protein, whereas 39 unique HTL peptides were identified from the Envelope protein and 23 unique HTL peptides from the NS5 protein. For CTL and HTL epitopes, each unique peptide sequence was counted only once, despite its predicted binding to multiple HLA alleles. The complete epitope prediction files, including the corresponding sequences, positions, HLA alleles, and predicted binding scores, are provided in Supplementary Material S2. Following the screening workflow, two linear B-cell epitopes originating from the Envelope protein met all predefined selection criteria (Table 2). Each peptide was predicted to be antigenic, non-allergenic and non-toxic and was therefore retained for inclusion in the final multi-epitope vaccine.

In addition, six CTL epitopes were obtained from the Envelope protein and NS5 protein after applying multiple selection parameters, including antigenicity, safety, and predicted HLA-binding performance (Table 3). These epitopes fulfilled the predefined selection requirements by combining predicted antigenicity with non-allergenic and non-toxic profiles, supporting their inclusion in the multi-epitope vaccine. The shortlisted CTL epitopes showed favorable predicted binding to multiple HLA class I molecules, consistently producing percentile rank values below 1. These predicted binding characteristics indicate favorable HLA class I-binding potential across the corresponding HLA alleles, supporting their consideration as candidate CTL epitopes for further experimental investigation. However, these predicted percentile ranks do not establish antigen processing, HLA presentation, or CTL activation, which require experimental validation.

Following the screening workflow, three HTL peptides from the Envelope protein and NS5 protein were retained (Table 4). All three peptides were predicted to be antigenic, non-allergenic, and non-toxic. All selected candidates showed favorable predicted binding to multiple HLA class II molecules and achieved percentile ranks below 10, supporting their selection as candidate HTL epitopes for further experimental investigation of CD4^+^ T-cell responses. These antigenicity, allergenicity, toxicity, and HLA-binding results represent computational predictions and do not constitute experimental evidence of antigen presentation or immunogenicity and require experimental validation.

Sequence cross-reactivity analysis of the selected vaccine epitopes was performed against *Homo sapiens* reference proteins using BLASTp. No significant sequence similarities were detected under the applied E-value threshold. However, IPISIVASL, VQKLGYILR, and KIPISIVASLSDLTP showed 100% sequence identity over 7-amino-acid aligned regions, with E-values of 0.24, 0.12, and 0.75, respectively. These short partial alignments were restricted to 7-amino-acid regions and did not represent complete sequence matches across the corresponding epitopes. Because the observed E-values were above the predefined significance threshold, these partial similarities were not considered significant BLASTp matches. Such short-region similarities may occur in short peptide sequences because of their limited sequence length. Overall, no complete or significant sequence similarity was identified between the selected epitopes and the human reference proteins under the applied BLASTp conditions. Detailed BLASTp results are provided in Supplementary Material S3.

### 3.3. Epitope Conservancy Analysis

The conservancy of the selected epitopes was evaluated across 200 homologous protein sequences represented by their respective accession numbers. ALPWTSPASSNWRNRE exhibited a 100% identity match in 195 of 200 sequences, corresponding to 97.50% of the analyzed sequences, while the remaining 5 sequences showed 93.75% identity. YIVVGRGDKQINHHWH showed 100% identity in 199 of 200 sequences, corresponding to 99.50%, while only 1 sequence showed 93.75% identity. Among the selected epitopes, GLMGALLLW was completely conserved across all 200 analyzed sequences, showing 100% identity in 200 of 200 sequences. In contrast, IPISIVASL showed comparatively greater sequence variation, with 100% identity in 93 of 200 sequences, while 106 sequences showed 88.89% identity and 1 sequence showed 77.78% identity. Similarly, KIPISIVASLSDLTP exhibited 100% identity in 93 of 200 sequences, whereas 106 sequences showed 93.33% identity and 1 sequence showed 53.33% identity. Finally, PPFGDSYIVVGRGDK showed 100% identity in 197 of 200 sequences, corresponding to 98.50%, with the remaining 3 sequences showing 93.33% identity.

For the NS5 protein, all five selected epitopes were completely conserved across the 200 analyzed sequences. FMWLGARFL, NALHFLNSM, VQKLGYILR, WLGARFLEF, and KLGEFGKAKGSRAIW each showed 100% identity in all 200 sequences, corresponding to 100% of the analyzed sequences. No sequence showed reduced identity for any of the selected epitopes from the NS5 protein.

Overall, GLMGALLLW and all five selected epitopes from the NS5 protein were the most highly conserved, showing 100% identity across all 200 analyzed sequences. YIVVGRGDKQINHHWH and PPFGDSYIVVGRGDK also showed very high conservation, with exact matches in 199 and 197 sequences, respectively, while ALPWTSPASSNWRNRE was identical in 195 sequences. In comparison, IPISIVASL and KIPISIVASLSDLTP showed greater sequence variation, with exact 100% matches in only 93 of the 200 analyzed sequences. The complete epitope conservancy results for both proteins, including accession numbers and identity percentages for each selected epitope, are provided in Supplementary Material S4.

### 3.4. Evaluation of Epitope Population Representation

**Figure 3:**
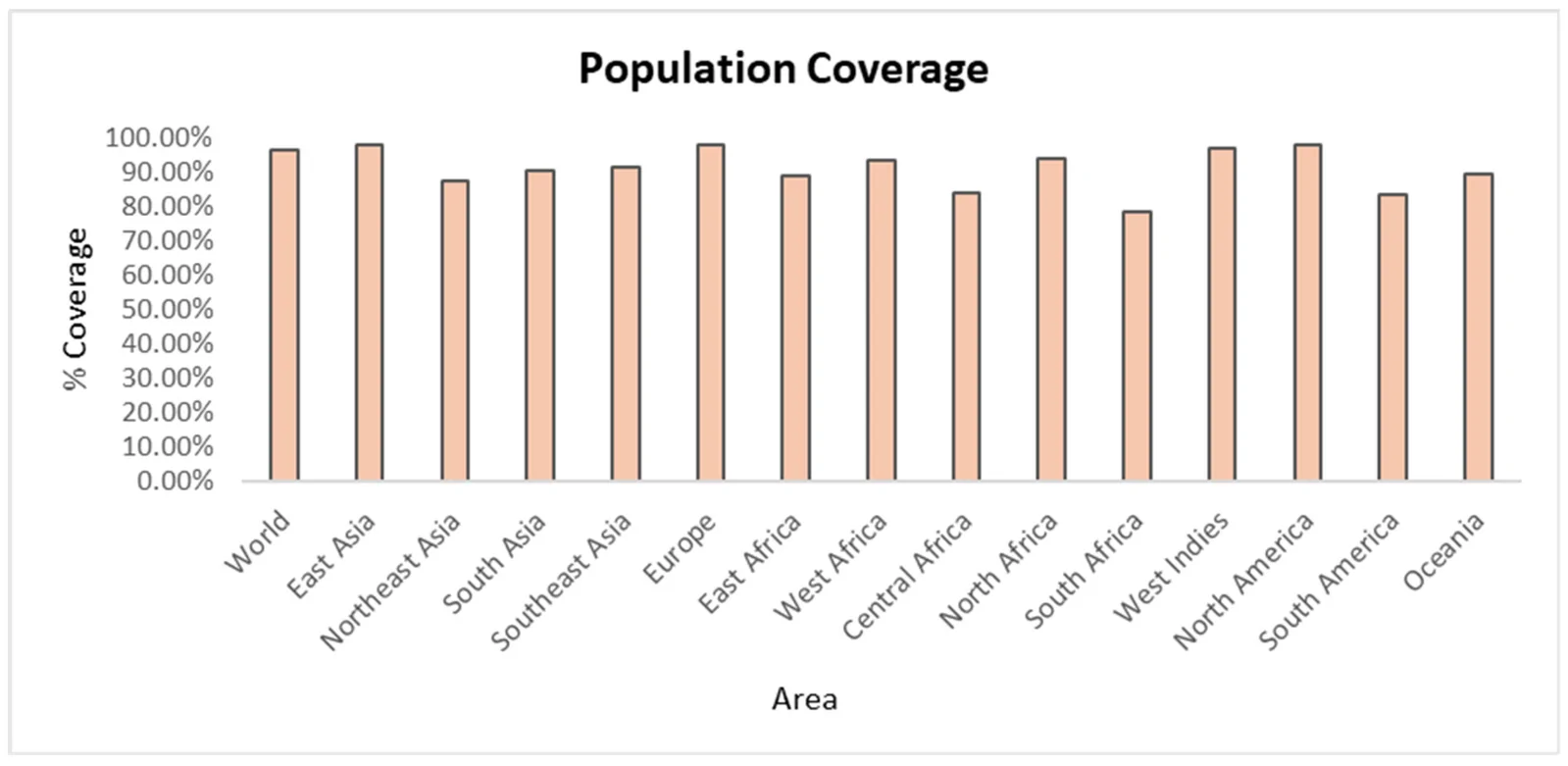
Geographic distribution of the predicted population coverage of the selected CTL and HTL epitopes based on their associated HLA alleles, as calculated using the IEDB Population Coverage Tool.

The combined population coverage of the retained CTL and HTL epitopes was estimated using the IEDB Population Coverage Tool. The selected CTL and HTL epitopes showed a predicted global population coverage of 96.41% (Figure 3). Regional variation was observed in the predicted population coverage, with the highest values recorded in North America (98.25%), Europe (98.19%), and East Asia (98.18%). Coverage was also high in the West Indies (97.19%), North Africa (94.33%), and West Africa (93.55%). Intermediate values were observed in Southeast Asia (91.42%), South Asia (90.71%), and East Africa (88.95%), whereas lower values were observed in Northeast Asia (87.73%), Central Africa (84.23%), South America (83.61%), and Oceania (89.41%). The lowest predicted coverage was observed in South Africa (78.58%). Overall, the selected epitopes showed substantial predicted HLA population coverage across the evaluated regions, although regional differences were evident. These population coverage estimates represent the proportion of individuals predicted to present one or more of the selected epitopes based on the evaluated HLA alleles and should not be interpreted as evidence of vaccine efficacy or clinical protection.

### 3.5. Multi-Epitope Vaccine Construction

**Figure 4:**
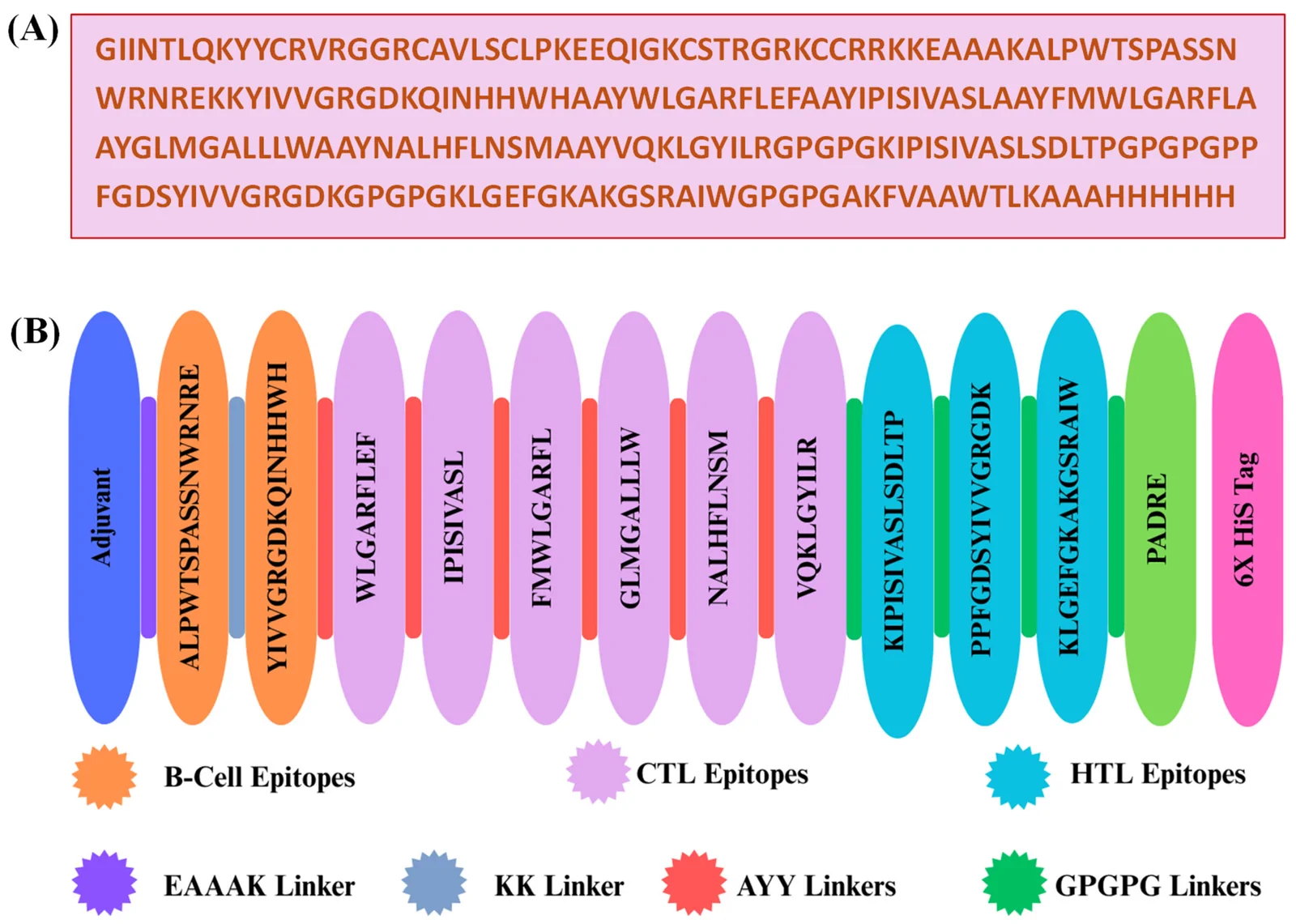
Amino acid sequence and schematic arrangement of the multi-epitope vaccine. (**A**) Amino acid sequence of the designed multi-epitope vaccine. (**B**) Schematic organization showing the β-defensin-3 adjuvant, selected B-cell, CTL, and HTL epitopes, linkers, PADRE sequence, and C-terminal 6×His tag.

The multi-epitope vaccine comprised the selected B-cell, CTL, and HTL epitopes arranged using the respective linkers, together with the β-defensin-3 adjuvant, PADRE sequence, and C-terminal 6×His tag (Figure 4). The C-terminal 6×His tag was incorporated to facilitate subsequent purification of the recombinant protein. Computational antigenicity evaluation classified the multi-epitope vaccine as a probable immunogen with a probability of 66%, while allergenicity analysis indicated no predicted allergenic potential.

### 3.6. Physicochemical Characterization

The physicochemical characteristics of the multi-epitope vaccine were evaluated to assess its predicted properties and suitability for recombinant production. Overall, the construct showed a low instability index, a high aliphatic index, and a negative GRAVY value. The SoluProt score was 0.861, which was above the threshold of 0.5 and indicated that the multi-epitope vaccine was predicted to be soluble. A complete summary of the physicochemical parameters is provided in Table 5.

### 3.7. Three-Dimensional Structure Prediction and Quality Assessment

**Figure 5:**
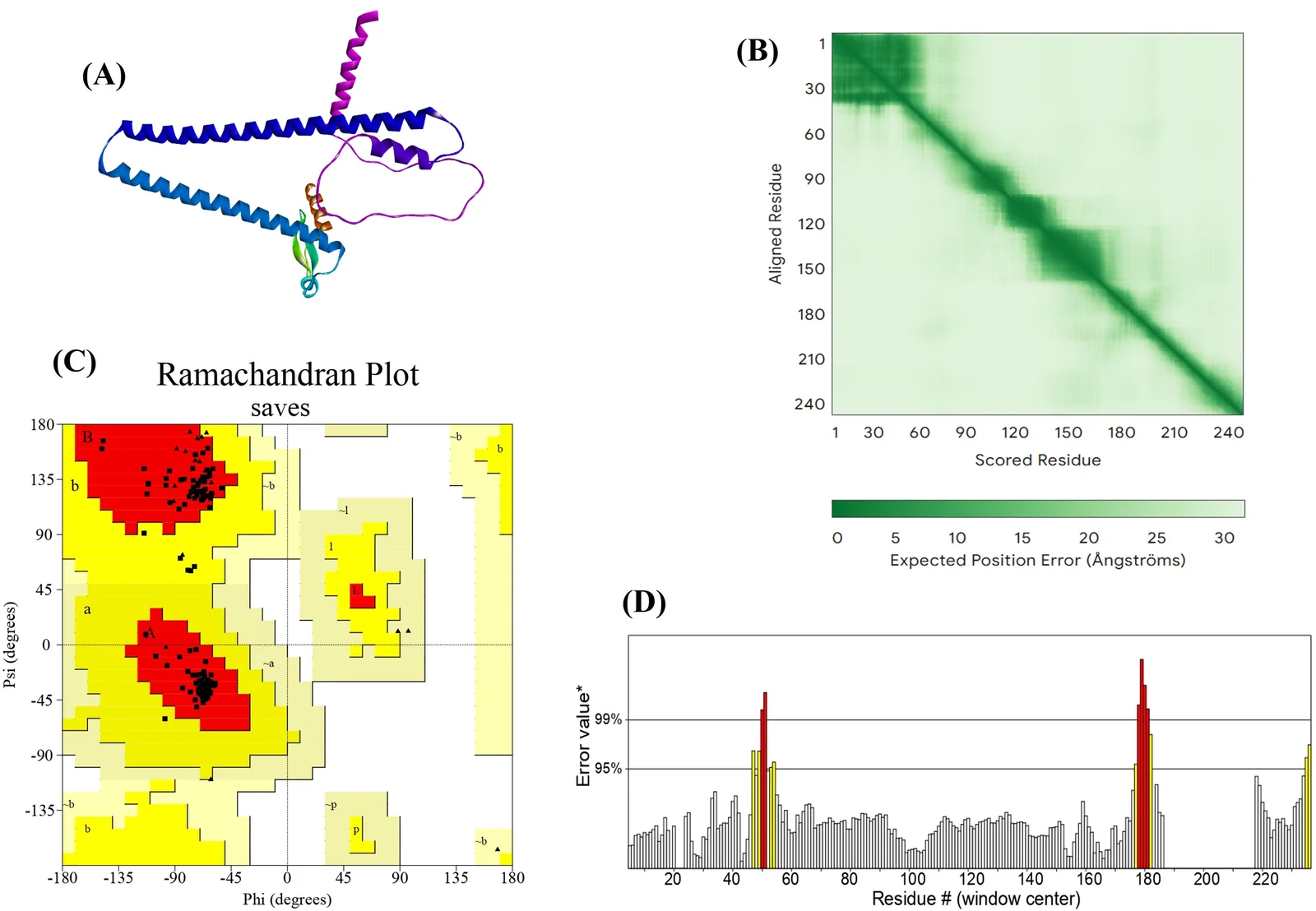
Three-dimensional structure prediction and quality assessment of the multi-epitope vaccine. (**A**) AlphaFold3 predicted structure of the multi-epitope vaccine, visualized using Discovery Studio. The color coding is provided by Discovery Studio to represent the cartoon structure. (**B**) Predicted aligned error (PAE) plot showing the predicted confidence between residue pairs, where darker green indicates lower expected position error and lighter green indicates higher expected position error. (**C**) Ramachandran plots showing the distribution of residues in favored, additionally allowed, generously allowed, and disallowed regions before and after structural refinement. Red regions represent the most favored regions, yellow regions represent the additionally allowed regions, light-yellow regions represent the generously allowed regions, and white regions represent the disallowed regions. In the Ramachandran plots, uppercase A, B, and L denote the core (most favored) α-helical, β-sheet, and left-handed α-helical regions, respectively; lowercase a, b, and l denote the corresponding additionally allowed regions, while p denotes the additionally allowed epsilon region. The symbols ~a, ~b, ~l, and ~p denote the corresponding generously allowed regions. Glycine residues are represented by triangular markers. (**D**) ERRAT quality profiles showing the overall quality factor of the vaccine structure before and after refinement. Gray bars represent regions with error values below the 95% rejection limit, yellow bars represent regions with error values between the 95% and 99% rejection limits, and red bars represent regions exceeding the 99% rejection limit.

The three-dimensional structure of the multi-epitope vaccine was predicted using AlphaFold3 (Figure 5A). The predicted aligned error (PAE) analysis showed the confidence of the predicted inter-residue distance relationships within the vaccine model (Figure 5B), while the pTM score obtained from the AlphaFold3 Server was 0.20. The resulting model was subsequently refined to improve its stereochemical properties before structural assessment. Ramachandran plot analysis (Figure 5C) showed that, before refinement, 88.1% of residues were located in favored conformational regions and 11.9% in additionally allowed regions, with none assigned to generously allowed or disallowed conformations. Following refinement, the proportion of residues within favored regions increased to 98.4%, whereas the remaining 1.6% were confined to additionally allowed regions, with no residues appearing in disallowed conformations. Independent assessment using ERRAT (Figure 5D) showed an increase in the overall quality factor from 85.34% before refinement to 92.86% after refinement. Overall, the refinement improved the Ramachandran and ERRAT results, whereas the PAE and pTM results indicated that the predicted structure should be interpreted with caution. Experimental validation is still needed to confirm these computational structural predictions.

### 3.8. Structure-Based Mapping of Discontinuous B-Cell Epitopes

**Figure 6:**
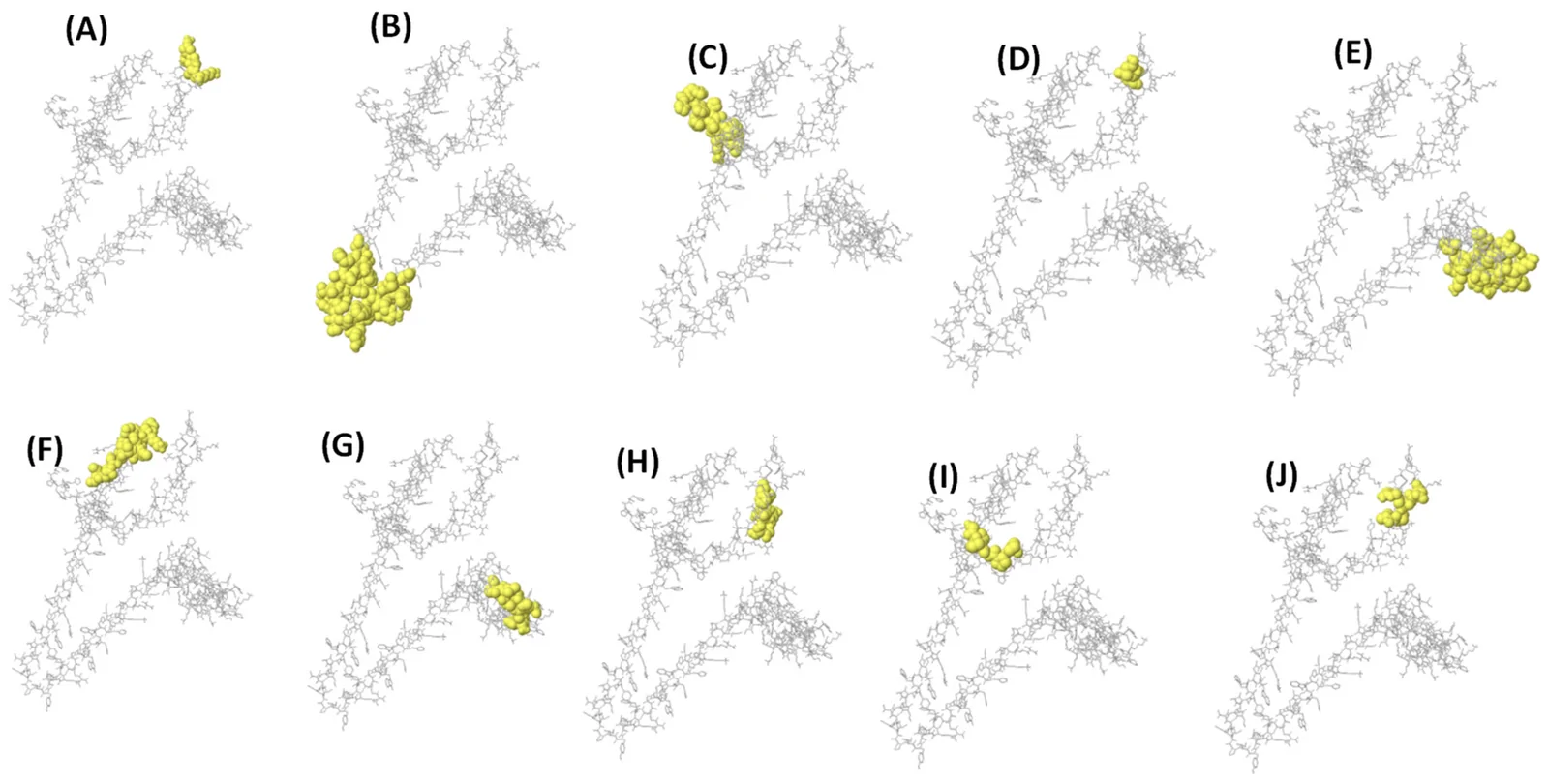
Predicted discontinuous B-cell epitope regions in the three-dimensional structure of the multi-epitope vaccine identified using ElliPro. Yellow color corresponds to the predicted discontinuous B-cell epitopes on the multi-epitope vaccine. (**A**) Epitope 1. (**B**) Epitope 2. (**C**) Epitope 3. (**D**) Epitope 4. (**E**) Epitope 5. (**F**) Epitope 6. (**G**) Epitope 7. (**H**) Epitope 8. (**I**) Epitope 9. (**J**) Epitope 10. Yellow regions indicate the predicted discontinuous B-cell epitopes.

Ten discontinuous B-cell epitope regions were identified in the predicted three-dimensional structure of the multi-epitope vaccine (Figure 6). These regions correspond to spatially clustered residues positioned on the predicted protein surface, suggesting their potential accessibility for antibody recognition. The predicted epitopes exhibited protrusion index values ranging from 0.534 to 0.836, indicating differences in the degree of residue protrusion from the protein surface. Among these, the top-ranked epitopes displayed scores of 0.836, 0.835, and 0.830, indicating greater residue protrusion within the predicted epitope regions. Additional regions showed moderate scores between 0.72 and 0.631, while smaller clusters presented lower values ranging from 0.561 to 0.534. The lowest-ranked clusters produced protrusion index values between 0.534 and 0.561. Their spatial arrangement indicates that multiple predicted exposed residue clusters are distributed across the protein surface. Overall, the structural analysis identified several surface-exposed predicted epitope regions that may serve as potential antibody-recognition sites.

### 3.9. Evaluation of Vaccine–Receptor Interactions

Molecular docking was performed to assess the predicted interactions of the multi-epitope vaccine with Toll-like receptors TLR2 and TLR4. For TLR2, cluster 0 contained 59 conformations, with model 0 showing a weighted ClusPro score of −1326.1. For TLR4, cluster 0 contained 30 conformations, and model 0 showed a weighted ClusPro score of −1230.2. These values represent weighted computational docking scores generated by ClusPro and were used for comparative assessment of the predicted vaccine–receptor complexes rather than as experimentally determined binding energies. The TLR2 docking generated a larger top-ranked cluster than the TLR4 docking, and the TLR2–vaccine complex was therefore selected for detailed interaction analysis and subsequent molecular dynamics simulation. The predicted docking interactions were interpreted as computational findings and do not constitute experimental evidence of receptor binding.

**Figure 7:**
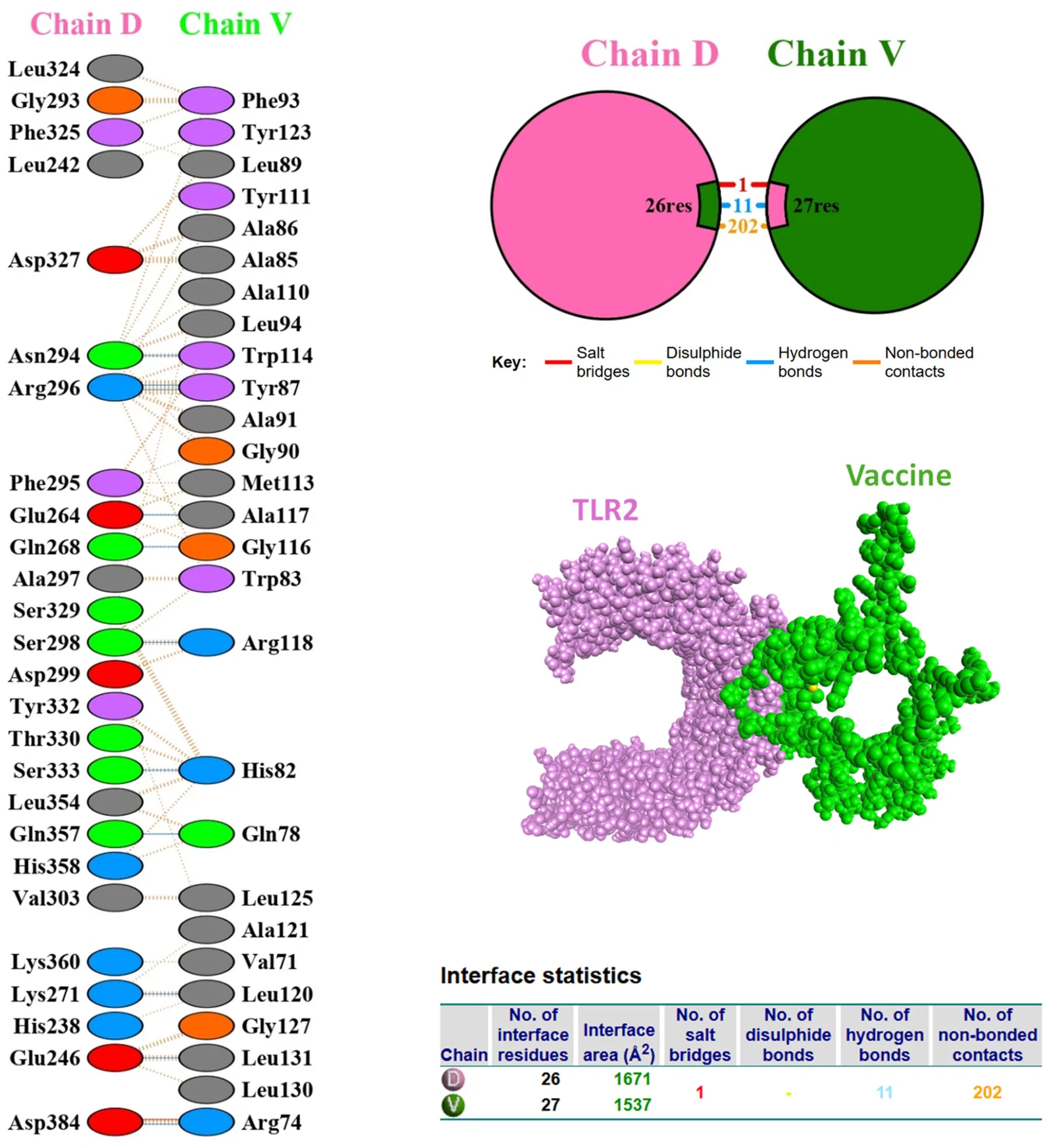
Structural and interaction analysis of the TLR2–vaccine complex, showing the residue contact map, three-dimensional complex structure, interface statistics, and intermolecular interactions, including salt bridges, hydrogen bonds, disulfide bonds, and non-bonded contacts.

Cluster 0 was selected for detailed interaction analysis because it contained the largest number of members, 59, and the corresponding TLR2–vaccine intermolecular interface was subsequently characterized. The contact surface involved 26 amino acid residues from TLR2 (Chain D) together with 27 residues from the vaccine (Chain V), producing interface areas of 1671 Å^2^ and 1537 Å^2^, respectively. The interface comprised 1 salt bridge, 11 hydrogen bonds, and 202 non-bonded contacts. Hydrogen-bond interactions involved vaccine residues including Trp114, Tyr87, Ala117, Gly116, Arg118, His82, Gln78, Leu120, Leu131, and Arg74, and TLR2 residues including Asn294, Arg296, Glu264, Gln268, Ser298, Ser333, Gln357, Lys271, Glu246, and Asp384 (Figure 7). Alongside these polar contacts, multiple hydrophobic and other non-covalent interactions were also detected, contributing additional intermolecular packing across the receptor–vaccine interface. Overall, the docking analysis identified an extensive network of intermolecular contacts at the TLR2–vaccine interface, including hydrogen bonds, a salt bridge, hydrophobic interactions, and other non-covalent contacts.

To further characterize the molecular interface of the selected TLR2–vaccine complex, COCOMAPS 2.0 analysis was performed. The complex exhibited a buried area of 3198.0 Å^2^, corresponding to an interface area of 1599.0 Å^2^, with 6.95% of the total surface area buried upon complex formation. The interface comprised 66.64% non-polar buried area (1065.65 Å^2^) and 33.36% polar buried area (533.4 Å^2^). A total of 73 interacting residues were identified, comprising 37 residues from TLR2 and 36 residues from the multi-epitope vaccine (Table 6).

**Figure 8:**
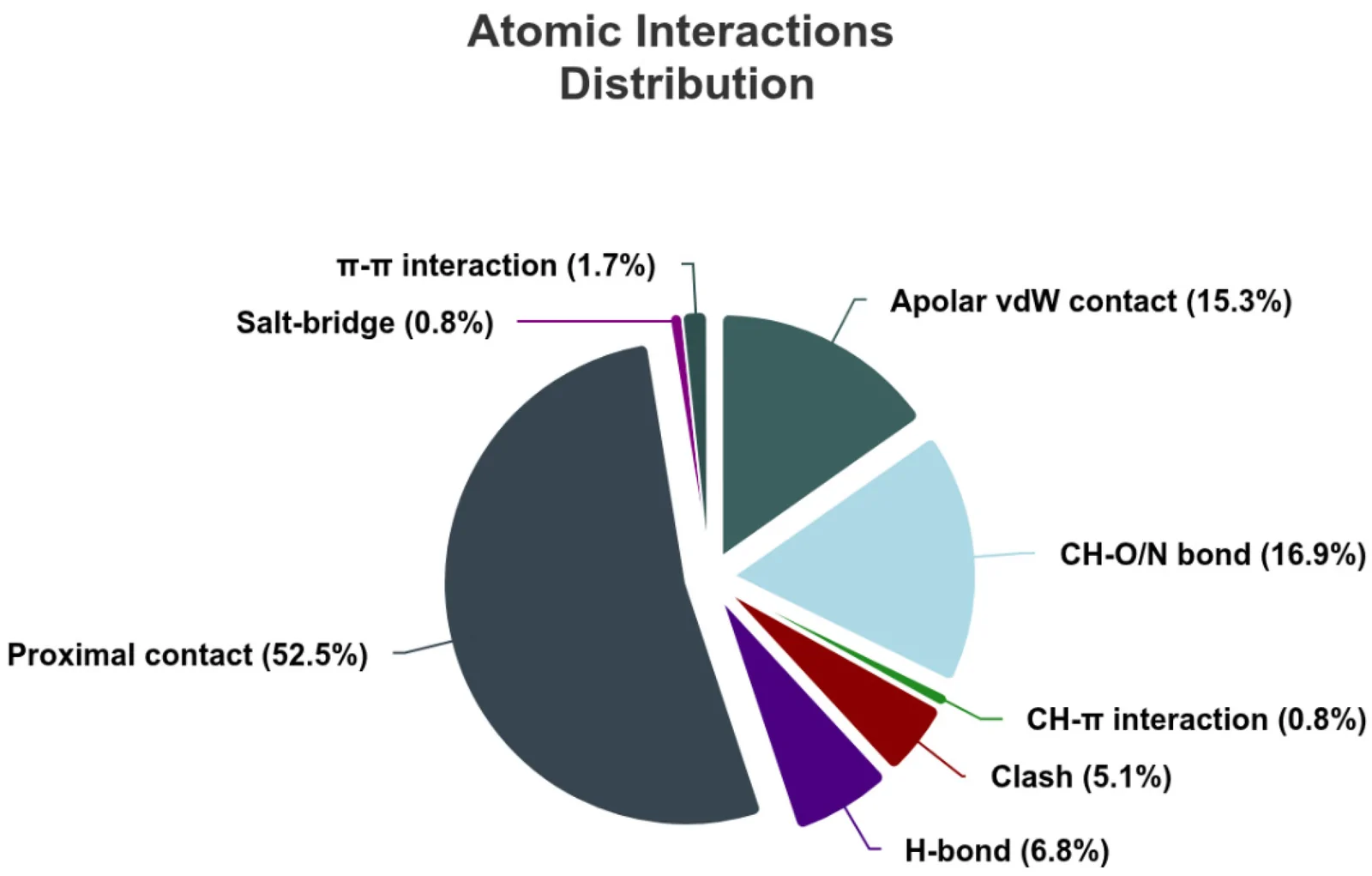
Distribution of atomic interactions at the TLR2–vaccine interface identified using COCOMAPS2.0, including proximal contacts, CH–O/N bonds, apolar van der Waals contacts, hydrogen bonds, clashes, π–π interactions, salt bridges, and CH–π interactions.

At the atomic level, 62 proximal contacts represented the largest interaction category, accounting for 52.5% of the interaction distribution (Figure 8). These were followed by 20 CH–O/N bonds (16.9%) and 18 apolar van der Waals contacts (15.3%). Additionally, 8 hydrogen bonds (6.8%), 6 clashes (5.1%), 2 π–π interactions (1.7%), 1 salt bridge (0.8%), and 1 CH–π interaction (0.8%) were identified. The interaction percentages shown in Figure 8 were obtained directly from the COCOMAPS 2.0 analysis and were rounded by the tool, resulting in a displayed total of 99.9%. Thus, the analyzed TLR2–vaccine interface comprised multiple intermolecular contact types, with proximal contacts, CH–O/N bonds, and apolar van der Waals contacts representing the predominant interaction categories. Overall, these results describe the composition of the TLR2–vaccine interface and the different types of atomic interactions identified within the docked complex.

### 3.10. Molecular Dynamics Simulations

**Figure 9:**
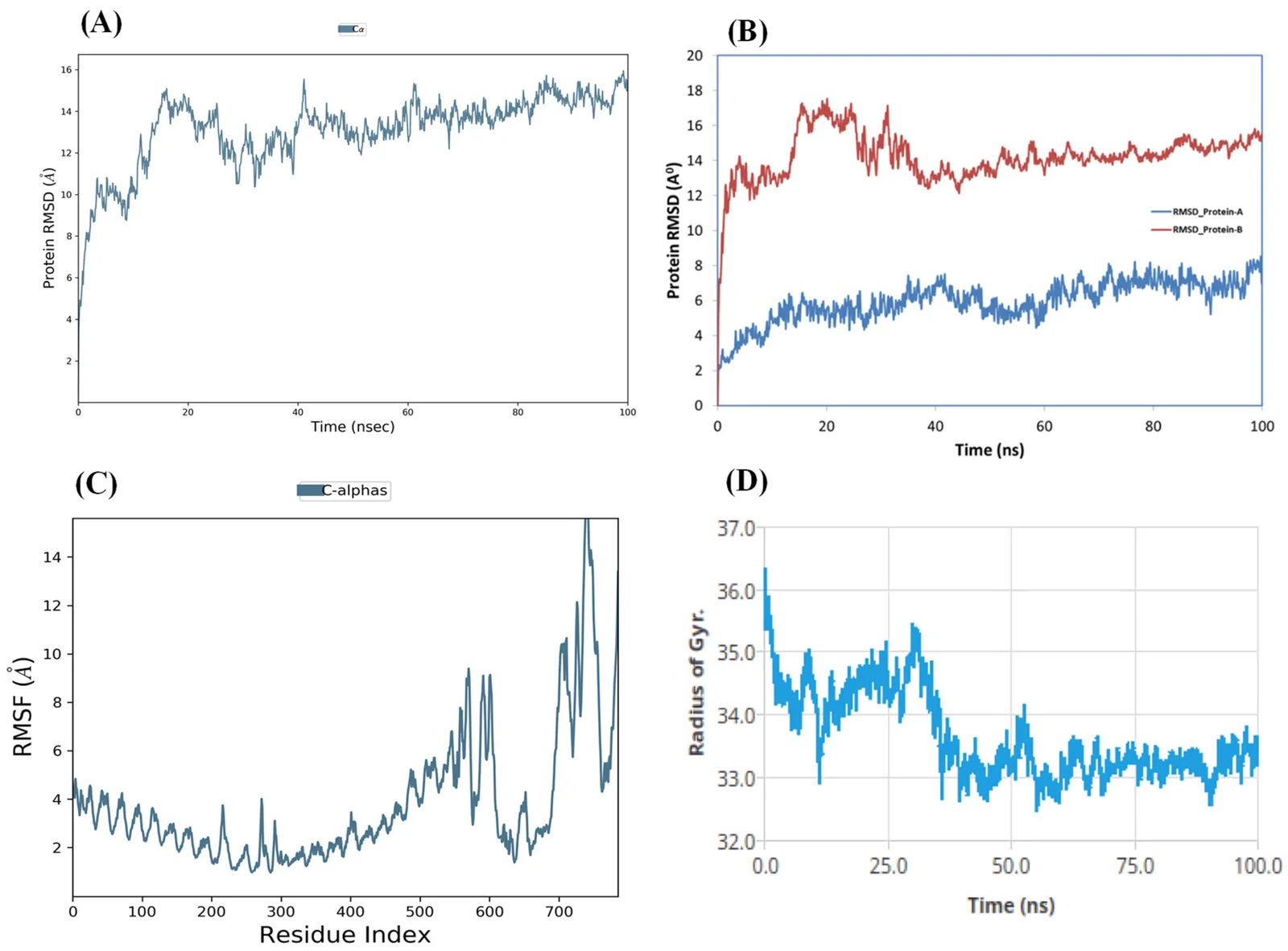
Molecular dynamics evaluation of the TLR2–vaccine complex over 100 ns. (**A**) RMSD profile of the complete TLR2–vaccine complex. (**B**) Separate RMSD profiles of TLR2 (Protein A) and the multi-epitope vaccine (Protein B). (**C**) RMSF profile showing residue-level fluctuations across TLR2 and the multi-epitope vaccine. (**D**) Radius of gyration (Rg) profile showing changes in the overall compactness of the complex during the simulation.

The 100 ns molecular dynamics trajectory was analyzed to examine the conformational behavior of the TLR2–vaccine complex. The RMSD profile of the overall complex (Figure 9A) showed an initial increase during the first 15–20 ns, followed by fluctuations between approximately 12 and 16 Å during the remainder of the simulation. This pattern reflects continued conformational adjustment of the complex during the simulation. The comparative RMSD analysis of the individual components (Figure 9B) showed that TLR2 (Protein A) exhibited lower deviation values, fluctuating between approximately 4 and 8 Å, whereas the multi-epitope vaccine (Protein B) displayed higher RMSD values. This difference indicates greater conformational flexibility of the vaccine component relative to TLR2 during the simulated trajectory. The higher RMSD observed for the multi-epitope vaccine may be related to its composition of multiple peptide sequences connected through flexible linker regions. These linkers provide conformational freedom to the connected peptide segments, which may contribute to the higher RMSD values observed for the vaccine component during the simulation.

The RMSF profile (Figure 9C) showed distinct differences in residue-level fluctuations between the two components. Residues 1–546, corresponding to TLR2, generally exhibited lower fluctuations, predominantly within 1–4 Å. In contrast, residues 547–786, corresponding to the multi-epitope vaccine, displayed greater fluctuations, with several regions exceeding 6 Å and reaching approximately 14–15 Å, particularly toward the terminal region. The increased fluctuations observed within the vaccine region, particularly toward the terminal portions, are consistent with the greater conformational freedom of the peptide segments connected by flexible linkers. The flexible linker regions may permit increased local movement of the adjoining peptide sequences, thereby contributing to the higher RMSF values observed in the vaccine component. However, the RMSF profile alone does not establish the contribution of specific individual residues to the overall RMSD. Overall, the RMSF results indicate comparatively restricted residue-level mobility in TLR2 and greater conformational flexibility in the vaccine component.

The radius of gyration (Rg) profile (Figure 9D) decreased from approximately 36.5 Å at the beginning of the simulation to approximately 33 Å within the first 40 ns. Subsequently, the Rg values fluctuated within a narrower range of approximately 33–34 Å throughout the remaining trajectory, indicating that the overall compactness of the system remained within a relatively consistent range during the latter part of the simulation. Collectively, the RMSD, RMSF, and Rg profiles demonstrate differential conformational behavior between TLR2 and the multi-epitope vaccine, with greater flexibility observed in the vaccine component. Together, these analyses provide computational insight into the conformational behavior and compactness of the TLR2–vaccine complex throughout the 100 ns simulation.

**Figure 10:**
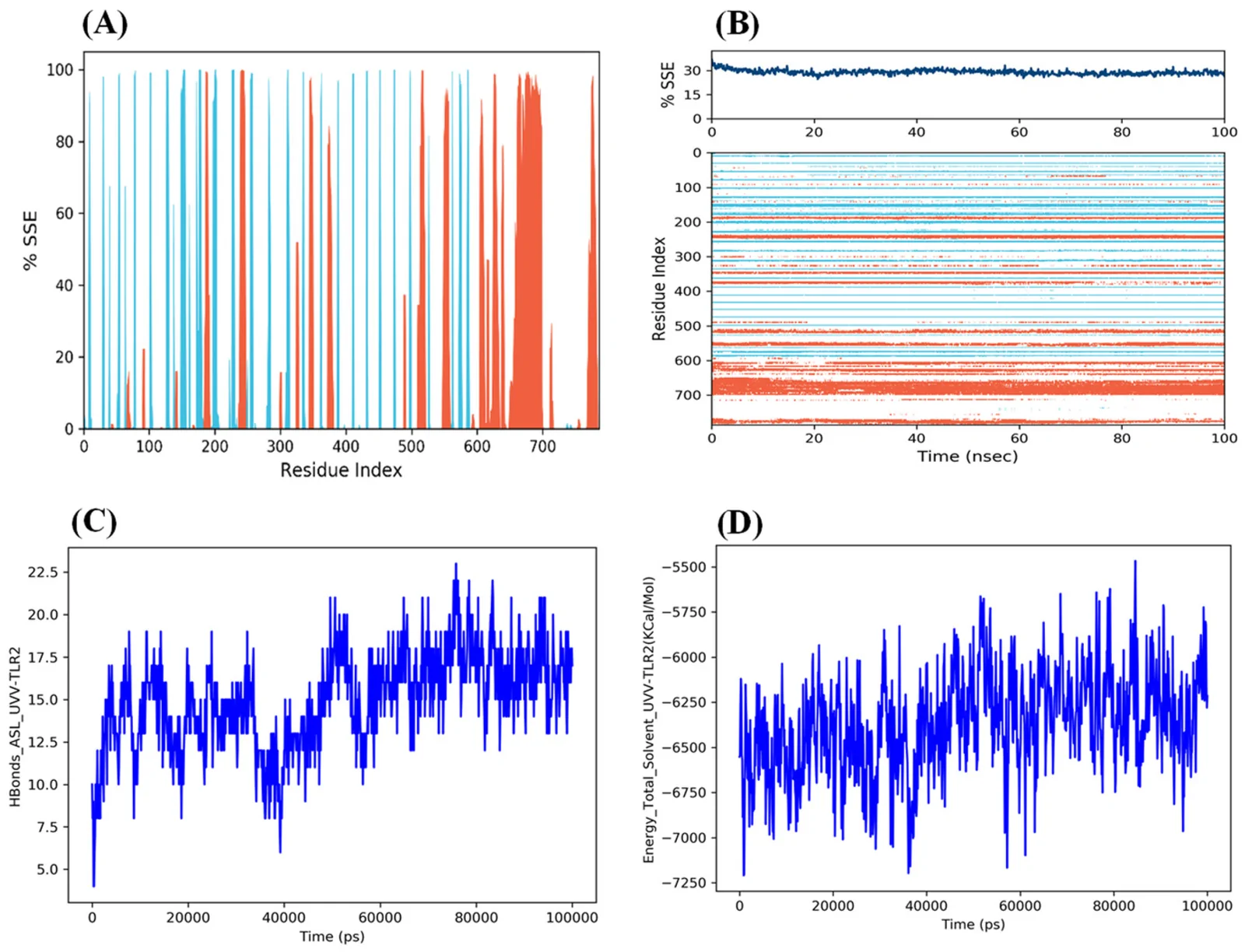
Molecular dynamics outputs for the TLR2–vaccine complex over 100 ns. (**A**) Residue-level secondary-structure assignment, where cyan represents α-helices and orange represents β-strand. (**B**) Overall secondary-structure composition (% SSE) throughout the simulation, with the corresponding residue-level secondary-structure map shown below, where cyan represents α-helices and orange represents β-strands. (**C**) Intermolecular hydrogen-bond interactions between TLR2 and the multi-epitope vaccine. (**D**) Solvent–complex energy profile during the simulation.

The structural behavior and energetic profile of the vaccine–TLR2 complex were further evaluated through analyses of secondary-structure evolution, intermolecular hydrogen-bond interactions, and energy fluctuations. The secondary-structure assessment (Figure 10A,B) indicated that the proportion of structural elements (SSE) remained relatively consistent throughout the 100 ns simulation, varying within the range of 28–32%. Residue-level mapping showed that the TLR2 segment (residues 1–546) maintained comparatively consistent secondary-structure characteristics, whereas the vaccine portion (residues 547–786) exhibited comparatively greater conformational flexibility. These variations primarily involved intermittent changes in the assigned secondary-structure elements.

Evaluation of intermolecular hydrogen bonding (Figure 10C) revealed a rapid increase in bond formation during the initial stage of the simulation, increasing from approximately 4–6 interactions to around 12–16 within the first 10 ns. Subsequently, the hydrogen-bond count fluctuated between 12 and 20, with transient increases to approximately 22 interactions. The observed intermolecular hydrogen bonds indicate the presence of TLR2–vaccine contacts throughout the simulated trajectory. However, these computational observations do not establish experimentally confirmed receptor binding or interaction stability. The solvent–complex energy (Figure 10D) remained within approximately −7200 to −5600 kcal/mol throughout the simulation, with fluctuations within this range and without an abrupt overall transition. This energy profile provides an additional computational descriptor of the simulated complex and shows that the energy values remained within a defined range throughout the trajectory.

The binding energetics of the TLR2–vaccine complex were estimated using the MM-GBSA approach, yielding a calculated ΔG_bind of −74.78 kcal/mol. This value represents the MM-GBSA estimate calculated from the final frame of the 100 ns molecular dynamics trajectory. Decomposition of the energy components showed that Coulombic interactions contributed −382.90 kcal/mol, while van der Waals interactions contributed −111.82 kcal/mol. Additional energy contributions included hydrogen bonding at −7.58 kcal/mol and lipophilic interactions at −32.03 kcal/mol (Table 7). These values represent selected computational energy components obtained from the selected trajectory frame and were used to characterize the energetic contributions within the vaccine–TLR2 complex.

The 100 ns molecular dynamics simulation revealed an initial phase of structural adjustment in the TLR2–vaccine complex, followed by continued conformational variation throughout the simulation period. Analysis of RMSD and radius of gyration profiles indicated that the overall system underwent structural rearrangement while maintaining its overall compactness during the latter part of the trajectory. The TLR2 receptor exhibited comparatively lower fluctuations, whereas the multi-epitope vaccine showed greater flexibility, which may be related to the presence of flexible linker segments within the multi-epitope vaccine. Residue-level fluctuation patterns and variations in secondary structure further distinguished the comparatively lower mobility of the receptor from the greater flexibility of the vaccine component. Intermolecular hydrogen-bond analysis throughout the 100 ns trajectory demonstrated the presence of TLR2–vaccine contacts during the simulation. The MM-GBSA analysis provided a computational estimate of the binding energetics based on the final trajectory frame. Together, these analyses characterize the conformational and interaction behavior of the vaccine–TLR2 complex during the simulated trajectory, while experimental studies are needed to confirm the predicted receptor interaction and structural behavior.

### 3.11. Computational Immune Response Assessment

**Figure 11:**
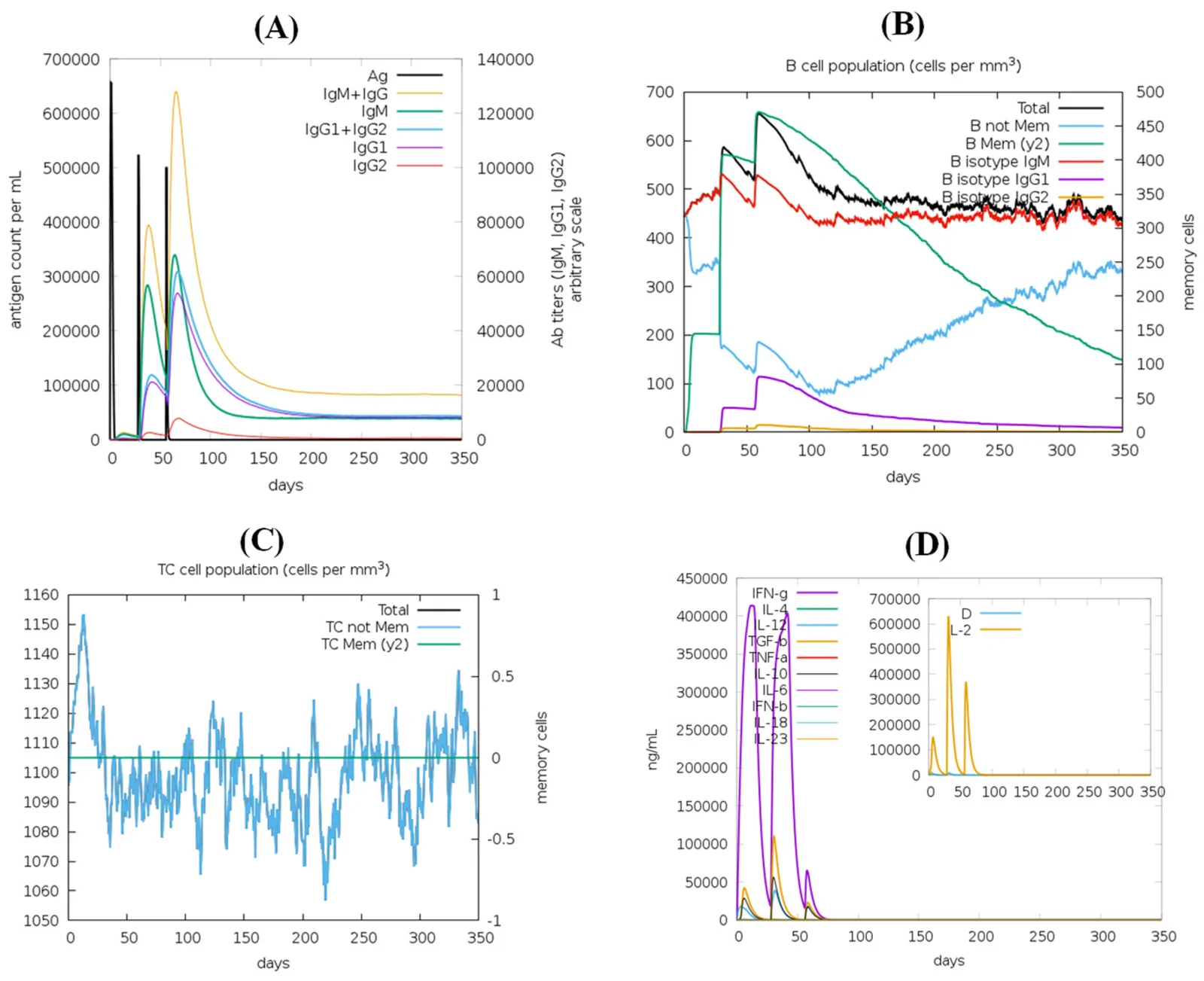
Predicted immune-response profiles of the multi-epitope vaccine during the 350-day simulated vaccination schedule using C-ImmSim. (**A**) Simulated antibody responses, including IgM and IgG subclasses. (**B**) Simulated B-cell population dynamics, including memory B-cell populations. (**C**) Simulated cytotoxic T-cell population dynamics. (**D**) Simulated cytokine profiles following vaccine administration.

The C-ImmSim analysis predicted the immune response to the multi-epitope vaccine during the simulated immunization regimen. The antibody response profile showed an increase in immunoglobulin concentrations following each simulated dose (Figure 11A). An initial primary response was observed after the first exposure, followed by higher simulated responses after the subsequent booster doses, with increases in IgM and IgG levels. Analysis of B-cell population kinetics showed corresponding changes in B-cell populations (Figure 11B), with memory B-cell populations remaining detectable throughout the simulation period. The simulated cytotoxic T-lymphocyte profile showed changes in the T-cell population following vaccine administration (Figure 11C). Cytokine profiling demonstrated changes in several immune mediators after immunization (Figure 11D), with IFN-γ and IL-2 showing increased simulated levels. Overall, the C-ImmSim results predicted enhanced humoral and cellular immune response patterns following the simulated booster administrations. These findings represent in silico predictions generated under the specified C-ImmSim simulation conditions and should be interpreted as computational predictions rather than experimental evidence of antibody production, memory-cell generation, cytotoxic T-cell activation, or cytokine-mediated immune responses. Experimental studies are required to validate the predicted immune-response patterns following administration of the proposed vaccine.

### 3.12. Codon Optimization and In Silico Cloning

**Figure 12:**
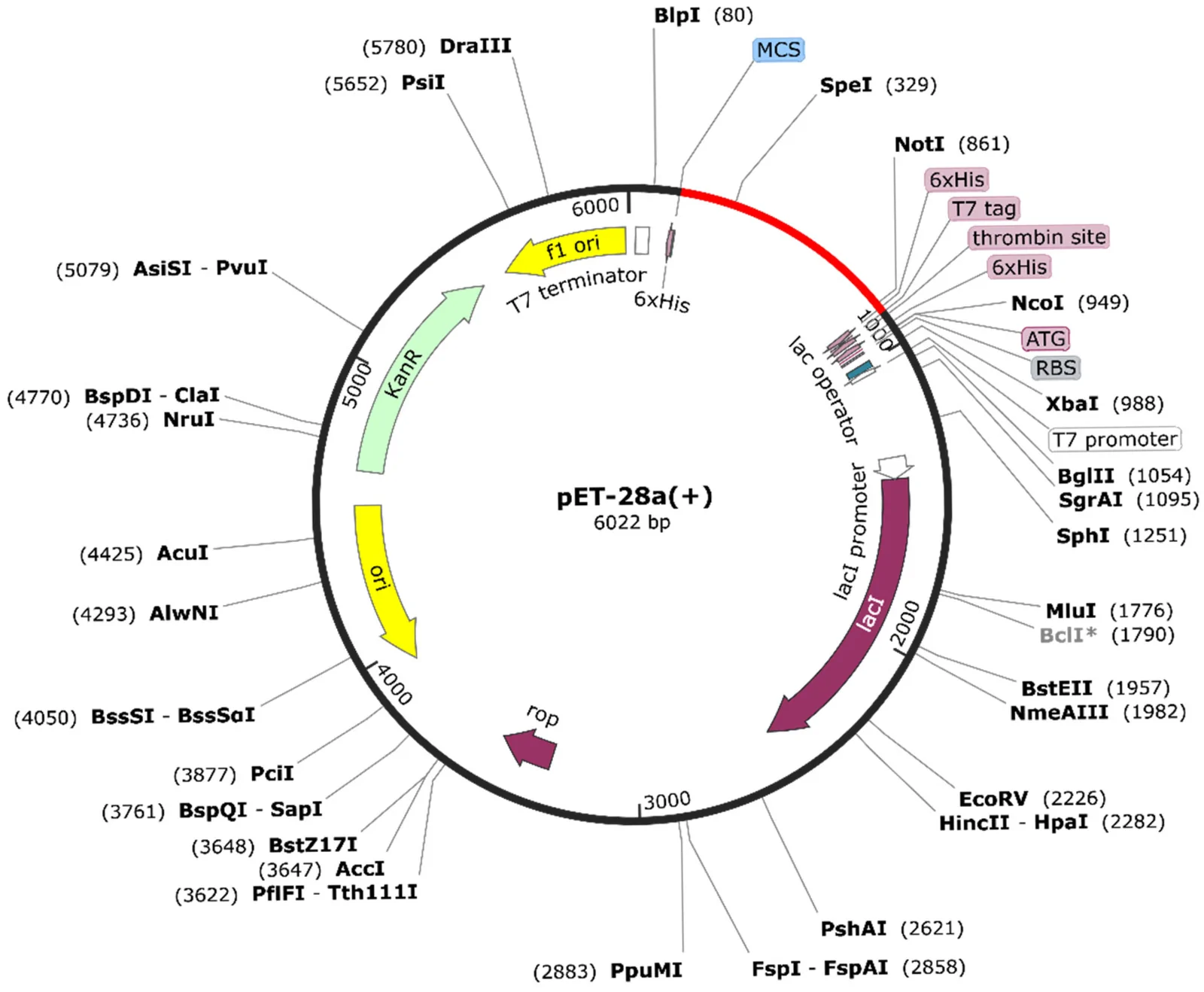
In silico cloning of the codon-optimized vaccine sequence into the pET-28a(+) expression vector, showing the orientation of the vaccine insert, restriction sites, vector backbone, and annotated genetic features. The asterisk (*) associated with the BclI restriction site denotes its Dam methylation sensitivity and is a standard restriction-site annotation on the pET-28a(+) vector designed by Novagen (Madison, WI, USA).

Codon optimization altered the nucleotide composition of the vaccine sequence, reducing the GC content from 65.20% to 59.65%, while the optimized sequence exhibited a CAI value of 0.73. The GC content and CAI were used as the principal parameters to evaluate codon adaptation toward *E. coli*. The CAI value of 0.73 indicated moderate codon adaptation toward the selected expression host. The optimized insert containing NdeI and XhoI restriction sites was positioned within the pET-28a(+) plasmid, as illustrated in Figure 12. The NdeI and XhoI restriction sites were correctly positioned for insertion of the vaccine sequence, and sequence translation predicted the maintenance of the correct reading frame. The cloning configuration was compatible with the pET-28a(+) expression system and retained the terminal 6×His-tag arrangement. The recombinant plasmid map displays the orientation of the inserted sequence together with the vector backbone and its annotated genetic features.

## 4. Discussion

This study designed a multi-epitope vaccine against Usutu virus by targeting Envelope protein and NS5 protein. The selection of these two proteins provided complementary targets for the vaccine design. The Envelope protein is a surface-exposed structural protein involved in host–cell attachment and membrane fusion, making it relevant to antibody recognition, whereas NS5 protein is an intracellular protein involved in viral genome replication and provides a distinct source of T-cell epitopes. Thus, the Envelope protein provides a target for antibody-mediated recognition, whereas NS5 protein provides a distinct source of targets for cellular immune responses. Similar design principles have also been reported in SARS-CoV-2 vaccine studies, where structural and non-structural proteins were considered together to broaden antigen selection [48].

The selected B-cell, CTL, and HTL epitopes showed favorable predicted antigenic characteristics and lacked predicted allergenicity and toxicity, supporting their selection for the multi-epitope vaccine, as a similar approach has been reported in previous studies [49]. The selected epitopes were also examined for sequence similarity with human proteins as an initial computational assessment of potential cross-reactivity. No complete sequence match was identified with human reference proteins. However, IPISIVASL, VQKLGYILR, and KIPISIVASLSDLTP showed 100% identity within 7-amino-acid regions, with E-values of 0.24, 0.12, and 0.75, respectively. These were partial matches, and experimental studies are required to assess their biological relevance.

The conservation analysis further supported the selection of the final epitopes. Most of the selected epitopes showed high conservation across the analyzed USUV sequences, with particularly high conservation observed among the NS5 protein derived epitopes. This conservation supports the selection of these regions as targets across the analyzed USUV sequence diversity. The high global population coverage of 96.41% obtained in this analysis indicates broad predicted HLA representation of the selected T-cell epitopes. However, regional differences in predicted population coverage indicate that HLA allele distribution can influence epitope representation among populations. Therefore, the 96.41% value represents predicted population coverage based on the evaluated HLA alleles and should not be interpreted as uniform coverage across all populations. These findings are consistent with reports on Chandipura and Nipah virus vaccine designs, where emphasis on HLA-binding diversity and epitope promiscuity was associated with extensive predicted population coverage [50,51]. It should be noted that the antigenicity, allergenicity, toxicity, and HLA-binding properties used for epitope selection were computationally predicted and therefore do not establish experimental antigen presentation or immunogenicity. The predicted HLA-binding characteristics should not be considered direct evidence of T-cell activation or protective immune responses and require experimental validation.

The inclusion of linker sequences AAY, GPGPG, and KK, together with the β-defensin-3 adjuvant and PADRE sequence, provided the selected components within the multi-epitope vaccine. Comparable linker and adjuvant strategies have been reported in studies of Marburg virus and lumpy skin disease [52,53]. The predicted physicochemical properties, including the low instability index, relatively high aliphatic index, negative GRAVY value, and SoluProt score of 0.861, indicated computationally favorable characteristics for further evaluation of the construct for recombinant production. These findings are consistent with previous observations in goatpox virus vaccine studies [54]. Structural refinement improved the predicted stereochemical properties, similar to structural assessment approaches used in Newcastle disease virus vaccine design [55]. However, the vaccine is an artificial protein containing multiple epitopes, flexible linkers, β-defensin, PADRE, and a terminal 6×His tag. Such a composition can allow different regions of the construct to adopt different conformations, and the actual folding and accessibility of individual epitopes therefore need to be determined experimentally.

The predicted three-dimensional structure should be interpreted in the context of the multi-epitope composition of the vaccine, which contains multiple epitope sequences connected through flexible linker regions and fused with an adjuvant. These flexible regions may permit greater conformational freedom and may contribute to conformational variability in some portions of the construct, consistent with the higher fluctuations observed for the vaccine component during molecular dynamics simulation. The refined model was evaluated using Ramachandran and ERRAT analyses, while the PAE plot and pTM score (0.20) provided by AlphaFold3 supplied additional information on model confidence. However, these computational assessments cannot establish intrinsic disorder or the experimentally adopted conformation of the complete construct, and experimental structural characterization will be required for definitive confirmation.

Docking analyses predicted favorable interactions of the proposed vaccine with both TLR2 and TLR4, producing computationally favorable binding arrangements. Comparable observations have been reported in Ebola vaccine studies, where interactions with Toll-like receptors were associated with enhanced immune signaling pathways [56]. Examination of the binding interface identified a diverse collection of residue contacts, including hydrogen-bond and hydrophobic interactions, which contributed to the predicted receptor–vaccine interaction. The ClusPro scores represent computational docking scores and should not be interpreted as experimentally determined binding energies or direct evidence of receptor engagement. Evaluation of the simulation trajectories further indicated that the receptor–vaccine association was maintained under the simulated conditions, while allowing localized structural adjustments rather than extensive conformational disruption. Molecular dynamics simulation provided further characterization of the interaction, indicating conformational flexibility within the vaccine–receptor system. The maintained structural characteristics, together with localized flexibility in linker-associated regions, may be favorable for the intended vaccine architecture, although their effects on antigen processing and presentation require experimental validation. The predicted binding energy further supports the computational assessment of the vaccine–TLR2 interaction. However, these computational findings do not establish direct TLR2 binding or receptor activation and require experimental validation.

The computational analyses collectively provide preliminary evidence supporting the predicted immunological potential of the proposed vaccine, which requires experimental validation. The predictive nature of epitope identification, HLA-binding assessment, structural modeling, molecular docking, MD simulation, MM-GBSA analysis, and immune simulation limits the extent to which these findings can represent biological processes. These methods cannot fully account for antigen processing, protein folding, receptor behavior, cellular immune mechanisms, or other biological factors in vivo. Due to resource limitations, the molecular dynamics simulation was performed for 100 ns. Future studies should extend the simulation duration and include independent replicate simulations with different initial velocities to further assess the reproducibility of the observed molecular behavior. In addition, quantitative measures such as receptor–vaccine center-of-mass distance, interface contact persistence, and interface residue contact frequency should be evaluated to provide further characterization of the interaction dynamics. MD snapshots at specific time intervals were not retained during the analysis; therefore, COCOMAPS2.0 analysis of the receptor–vaccine interface was performed only for the docked complex and not for specific snapshots during the MD simulation. Future investigations should incorporate systematic interface analysis of representative MD snapshots to evaluate the temporal conservation of receptor–vaccine interactions, thereby providing a more comprehensive characterization of the interaction dynamics. Although the predicted outcomes are encouraging, the conclusions remain dependent on computational modeling and therefore cannot fully reproduce the complexity of biological systems. Experimental validation should include recombinant expression and purification of the multi-epitope vaccine, structural characterization, assessment of epitope processing and HLA presentation, evaluation of antigen-specific antibody and T-cell responses, investigation of TLR2-associated responses, and experimental assessment of cross-reactivity and safety. Subsequent in vivo studies should evaluate immunogenicity and protective performance against USUV.

## 5. Conclusions

The present study designed a computationally prioritized multi-epitope vaccine against Usutu virus using selected regions of the Envelope protein and NS5 protein. The selected epitopes showed favorable predicted antigenicity, high conservation, broad predicted HLA population coverage, and suitable predicted physicochemical properties. Structural modeling, TLR interaction analysis, molecular dynamics simulation, MM-GBSA analysis, and immune simulation provided additional computational information supporting further investigation of the vaccine candidate. However, this study was conducted entirely using in silico methods, and the findings represent computational predictions rather than experimentally confirmed results. Further experimental validation is required to verify the predicted properties and determine whether the observed results can be reproduced under biological conditions. Experimental validation should include recombinant protein expression and purification, structural characterization, epitope processing and HLA presentation, immune recognition, TLR2-associated responses, cross-reactivity and safety assessment, followed by in vivo evaluation of immunogenicity and protective performance. The proposed construct should therefore be considered a computationally prioritized vaccine candidate, with its biological and protective potential requiring laboratory and preclinical validation.

## Figures and Tables

**Table 1:** Predicted biological and immunological features of the selected Usutu virus proteins.

Protein Name	Uniprot Id	Function	Antigenicity	Allergenicity	Subcellular Localization		
					Outside	Inside	TMhelix
Envelope protein	A0A7S5IEW7	Viral surface glycoprotein responsible for host–cell attachment, membrane fusion, and viral entry	Probable immunogen with a probability of 66%	Non-allergen	1–386	410–427	387–409
NS5 protein	A0A075W8L0	RNA-dependent RNA polymerase involved in viral genome replication and RNA capping	Probable immunogen with a probability of 100%	Non-allergen	1–336	-	-

**Table 2:** Finalized linear B-cell epitopes.

Protein Name	Epitopes	Antigenicity	Allergenicity	Toxicity
Envelope protein	ALPWTSPASSNWRNRE	Probable immunogen with a probability of 66%	Non-allergen	Non-toxic
	YIVVGRGDKQINHHWH	Probable immunogen with a probability of 66%	Non-allergen	Non-toxic

**Table 3:** Finalized CTL epitopes.

Protein Name	Epitopes	Antigenicity	Allergenicity	Toxicity	Alleles	Percentile Rank Score
Envelope protein	GLMGALLLW	Probable immunogen with a probability of 66%	Non-allergen	Non-toxic	HLA-A*32:01	0.05
					HLA-B*58:01	0.27
					HLA-B*57:01	0.37
					HLA-A*23:01	0.75
	IPISIVASL	Probable immunogen with a probability of 66%	Non-allergen	Non-toxic	HLA-B*51:01	0.02
					HLA-B*07:02	0.04
					HLA-B*35:01	0.05
					HLA-B*53:01	0.05
					HLA-B*08:01	0.18
					HLA-A*68:02	0.84
NS5 protein	FMWLGARFL	Probable immunogen with a probability of 100%	Non-allergen	Non-toxic	HLA-A*02:01	0.24
					HLA-A*02:03	0.61
					HLA-A*02:06	0.79
	NALHFLNSM	Probable immunogen with a probability of 66%	Non-allergen	Non-toxic	HLA-B*35:01	0.3
					HLA-B*51:01	0.44
					HLA-A*26:01	0.92
					HLA-B*44:03	0.1
					HLA-B*40:01	0.11
					HLA-B*44:02	0.15
	VQKLGYILR	Probable immunogen with a probability of 100%	Non-allergen	Non-toxic	HLA-A*31:01	0.11
					HLA-A*30:01	0.62
					HLA-A*33:01	0.69
	WLGARFLEF	Probable immunogen with a probability of 66%	Non-allergen	Non-toxic	HLA-B*08:01	0.16
					HLA-A*24:02	0.9
					HLA-A*23:01	0.9

**Table 4:** Finalized HTL epitopes.

Protein Name	Epitopes	Antigenicity	Allergenicity	Toxicity	Alleles	Percentile Rank Score
Envelope protein	KIPISIVASLSDLTP	Probable immunogen with a probability of 66%	Non-Allergen	Non-Toxic	HLA-DRB1*04:05	0.45
					HLA-DRB1*07:01	5.3
					HLA-DRB1*04:01	5.9
					HLA-DRB1*09:01	8.8
					HLA-DRB1*15:01	9.6
	PPFGDSYIVVGRGDK	Probable immunogen with a probability of 66%	Non-Allergen	Non-Toxic	HLA-DRB1*11:01	7.7
NS5 protein	KLGEFGKAKGSRAIW	Probable immunogen with a probability of 66%	Non-Allergen	Non-Toxic	HLA-DRB1*01:01	3.5
					HLA-DRB1*09:01	7.6

**Table 5:** Predicted physicochemical characteristics of the multi-epitope vaccine.

Parameters	Results
Number of amino acids	240
Theoretical pI	10.11
Molecular weight	26,075.42 Da
Instability index	18.20
Aliphatic index	82.29
Grand average of hydropathicity	−0.113
Solubility	0.861

**Table 6:** Interface and atomic interaction characteristics of the TLR2–vaccine complex determined using COCOMAPS2.0.

Category	Parameter	Value
Interface characteristics	Buried area upon complex formation/Interface area (Å^2^)	3198.0/1599.0
	Buried area upon complex formation (%)	6.95
	Polar buried area upon complex formation/Interface area (Å^2^)	1066.8/533.4
	Polar interface (%)	33.36
	Non-polar buried area upon complex formation/Interface area (Å^2^)	2131.3/1065.65
	Non-polar interface (%)	66.64
Residue interactions	Total number of interacting residues	73
	Number of interacting residues in Chain 1 (TLR2)	37
	Number of interacting residues in Chain 2 (multi-epitope vaccine)	36
Atomic interactions	Number of proximal contacts	62
	Number of CH–O/N bonds	20
	Number of apolar van der Waals contacts	18
	Number of hydrogen bonds	8
	Number of clashes	6
	Number of π–π interactions	2
	Number of salt bridges	1
	Number of CH–π interactions	1

**Table 7:** MM-GBSA binding energy components of the vaccine–TLR2 complex.

Energy Component	Results (kcal/mol)
ΔG Bind	−74.78
Coulomb Energy	−382.90
van der Waals Energy	−111.82
Hydrogen Bond Energy	−7.58
Lipophilic Energy	−32.03

## Data Availability

The original contributions presented in this study are included in the article/Supplementary Material. Further inquiries can be directed to the corresponding author and Supplementary Materials S1–S5. Additional raw data, including the vaccine amino acid sequence, three-dimensional model, docked complexes, and molecular dynamics simulation data, are provided in Supplementary Material S5.

## Supplementary Materials

The following supporting information can be downloaded at: https://www.mdpi.com/article/10.3390/microorganisms14092026/s1. Supplementary Material S1: The 200 homologous protein sequences of the envelope and NS5 proteins identified using BLASTp. Supplementary Material S2: All predicted B-cell and T-cell epitopes. Supplementary Material S3: Results of the sequence cross-reactivity analysis of the selected vaccine epitopes against *Homo sapiens* reference proteins (taxid: 9606). Supplementary Material S4: Epitope sequence conservation analysis of all selected epitopes from the envelope and NS5 proteins used for vaccine design. Supplementary Material S5: Additional raw data from the study, including protein sequences, the vaccine amino acid sequence, the three-dimensional vaccine model, docking PDB files, and molecular dynamics simulation data.
