# Supplementary material for: Design and Evaluation of a Multi-Epitope Vaccine Targeting Conserved Envelope and NS5 Proteins of Usutu Virus Using Immunoinformatics
Source: Microorganisms. 2026 Sep 11;14(9):2026. doi: 10.3390/microorganisms14092026 (PMC13609697; doi:10.3390/microorganisms14092026)
Supplement: Supplementary file 1 [file microorganisms-14-02026-s001.zip › Supplementary Material_S2/B-Cell Epitopes/B-Cell Epitopes_Envelope protein.pdf]

Predicted linear B-cell epitopes from Envelope protein obtained using ABCPred

Rank	Sequence	Start position	Score
1	YGEISVECEPRNGLNT	118	0.99
2	GMSWITQGLMGALLLW	390	0.97
3	PKRAEDTYVCKSGVTD	18	0.92
4	CKSGVTDRGWGNGCGL	27	0.89
5	GIFIHGSTSSDTHGNY	75	0.88
5	SNCPTTGEAHNPKRAE	7	0.88
5	KGSIDTCANFTCSLKA	45	0.88
6	LTPIGRMVTANPYVAS	283	0.87
7	TTYSMCTEKFSFAKNP	234	0.86
8	RGWGNGCGLFGKGSID	34	0.84
8	ALPWTSPASSNWRNRE	157	0.84
8	TVKMGDYGEISVECEP	112	0.84
9	AALGDTAWDFGSVGGI	353	0.82
10	SSIGKAFITTIKGAQR	336	0.81
11	VGKAVHQVFGGAFRTL	372	0.80
12	TCSLKAMGRMIQPENV	55	0.79
12	PPFGDSYIVVGRGDKQ	311	0.79
12	HGTVVLELQYTGSDGP	254	0.79
13	SQAGRFTITPNSPAIT	97	0.78
13	SSQLGASQAGRFTITP	91	0.78
13	STSSDTHGNYSSQLGA	81	0.78
14	KVLVEMEPPFGDSYIV	304	0.77
14	REILLEFEEPHATKQS	171	0.77
15	ITTIKGAQRLAALGDT	343	0.76
15	SSGPKHLLVHREWFND	140	0.76
16	SFSGSVKLTSGHLKCR	208	0.75
17	GDKQINHHWHKAGSSI	323	0.74
17	YIVVGRGDKQINHHWH	317	0.74
17	SGHLKCRVKMEKLTLK	217	0.74
18	VFGGAFRTLFGGMSWI	379	0.73
18	DGPCKIPISIVASLSD	267	0.73
19	ANPYVASSEANAKVLV	292	0.72
19	GSQEGALHQALAGAVP	191	0.72
20	GLMGALLLWMGVNARD	397	0.71
20	NGLNTEAYYIRSSGPK	129	0.71
21	NPTDTGHGTVVLELQY	248	0.70
22	AGAVPVSFSGSVKLTS	202	0.69
23	LWMGVNARDRSIALVM	404	0.66
24	SVGGIFNSVGKAVHQV	364	0.64

25	EPHATKQSVVALGSQE	179	0.61
26	ENVKYEVGIFIHGSTS	68	0.60
27	HREWFNDLALPWTSPA	149	0.56
28	ARDRSIALVMLATGGV	410	0.55
29	SSEANAKVLVEMEPPF	298	0.54
29	PASSNWRNREILLEFE	163	0.54
