# Supplementary material for: Design and Evaluation of a Multi-Epitope Vaccine Targeting Conserved Envelope and NS5 Proteins of Usutu Virus Using Immunoinformatics
Source: Microorganisms. 2026 Sep 11;14(9):2026. doi: 10.3390/microorganisms14092026 (PMC13609697; doi:10.3390/microorganisms14092026)
Supplement: Supplementary file 1 [file microorganisms-14-02026-s001.zip › Supplementary Material_S3.pdf]

Sequence cross-reactivity analysis of the selected vaccine epitopes against Homo sapiens reference proteins (taxid: 9606) was performed using BLASTP.

Sr.No.	Epitope sequence	Human protein	Matching protein accession	Alignment length with subject (aa)	% Identity	E-value
1	**ALPWTSPASSNWRNRE**	Putative RNA-binding protein 15B	NP_037418.3	12	66.67%	0.63
2	**YIVVGRGDKQINHHWH**	phospholipid scramblase 2 isoform 3	NP_001382366.1	14	57.14%	3.6
3	**GLMGALLLW**	olfactory receptor 52N4	NP_001005175.3	9	77.78%	0.24
4	**IPISIVASL**	solute carrier family 46 member 2	NP_149040.3	7	100%	0.24
5	**FMWLGARFL**	Calcium-activated potassium channel subunit alpha-1 isoform c	NP_001154824.1	7	85.71%	2.8
6	**NALHFLNSM**	T-complex protein 1 subunit delta isoform a	NP_006421.2	8	87.50%	0.12
7	**VQKLGYILR**	macrophage mannose receptor 1 precursor	NP_002429.1	7	100%	0.12
8	**WLGARFLEF**	desmoplakin isoform I	NP_004406.2	13	61.54%	0.34
9	**KIPISIVASLSDLTP**	solute carrier family 46 member 2	NP_149040.3	7	100%	0.75
10	**PPFGDSYIVVGRGDK**	transducin-like enhancer protein 3 isoform b	NP_001098662.1	11	72.73%	1.1
11	**KLGEFGKAKGSRAIW**	zinc finger protein 185 isoform 10	NP_001382183.1	10	70%	0.75

Note: The percentage identity values reported by BLASTP refer only to the residues included in the specific aligned region and not to the complete epitope sequence. For example, a 100% identity reported for a 7-residue alignment means that all 7 of the 7 aligned residues matched the corresponding residues in the human reference protein, while the remaining 2 residues of the 9-residue epitope were not matched in that alignment.
