# Supplementary material for: Design and Evaluation of a Multi-Epitope Vaccine Targeting Conserved Envelope and NS5 Proteins of Usutu Virus Using Immunoinformatics
Source: Microorganisms. 2026 Sep 11;14(9):2026. doi: 10.3390/microorganisms14092026 (PMC13609697; doi:10.3390/microorganisms14092026)
Supplement: Supplementary file 1 [file microorganisms-14-02026-s001.zip › Supplementary Material_S4/Envelope Protein Sequence Conservation Analysis.pdf]

Supplementary Table S1. Sequence conservation of selected epitopes from Envelope protein across 200 protein sequences

Sr. No	Epitopes / Acession No.	ALPWTSPASSNWRNRE	YIVVGRGDKQINHHWH	GLMGALLLW	IPISIVASL	KIPISIVASLSDLTP	PPFGDSYIVVGRGDK
1	XRL80563.1	100.00%	100.00%	100.00%	100.00%	100.00%	100.00%
2	WVE62481.1	100.00%	100.00%	100.00%	100.00%	100.00%	100.00%
3	WQA41494.1	100.00%	100.00%	100.00%	100.00%	100.00%	100.00%
4	WPD49163.1	100.00%	100.00%	100.00%	100.00%	100.00%	100.00%
5	WQA41498.1	100.00%	100.00%	100.00%	100.00%	100.00%	100.00%
6	WQA41497.1	100.00%	100.00%	100.00%	100.00%	100.00%	100.00%
7	AID60201.1	100.00%	100.00%	100.00%	100.00%	100.00%	100.00%
8	YBL38152.1	100.00%	100.00%	100.00%	100.00%	100.00%	100.00%
9	YBL38141.1	100.00%	100.00%	100.00%	100.00%	100.00%	100.00%
10	YBL38133.1	100.00%	100.00%	100.00%	100.00%	100.00%	100.00%
11	YBL38089.1	100.00%	100.00%	100.00%	100.00%	100.00%	100.00%
12	YBL38012.1	100.00%	100.00%	100.00%	100.00%	100.00%	100.00%
13	WVE62480.1	100.00%	100.00%	100.00%	100.00%	100.00%	100.00%
14	WQA41506.1	100.00%	100.00%	100.00%	100.00%	100.00%	100.00%
15	WMP24385.3	100.00%	100.00%	100.00%	100.00%	100.00%	100.00%
16	WGU19507.1	100.00%	100.00%	100.00%	100.00%	100.00%	100.00%
17	WEM02436.1	100.00%	100.00%	100.00%	100.00%	100.00%	100.00%
18	AQM54950.1	100.00%	100.00%	100.00%	100.00%	100.00%	100.00%
19	AIN76231.1	100.00%	100.00%	100.00%	100.00%	100.00%	100.00%
20	AID60256.1	100.00%	100.00%	100.00%	100.00%	100.00%	100.00%
21	AID60232.1	100.00%	100.00%	100.00%	100.00%	100.00%	100.00%
22	YBL38143.1	100.00%	100.00%	100.00%	100.00%	100.00%	100.00%
23	YBL38140.1	100.00%	100.00%	100.00%	100.00%	100.00%	100.00%
24	YBL38007.1	100.00%	100.00%	100.00%	100.00%	100.00%	100.00%
25	XOF08382.1	100.00%	100.00%	100.00%	100.00%	100.00%	100.00%
26	WVE62479.1	100.00%	100.00%	100.00%	100.00%	100.00%	100.00%
27	WQA41503.1	100.00%	100.00%	100.00%	100.00%	100.00%	100.00%
28	WPD49160.1	100.00%	100.00%	100.00%	100.00%	100.00%	100.00%
29	WMP24437.3	100.00%	100.00%	100.00%	100.00%	100.00%	100.00%
30	QID21448.1	100.00%	100.00%	100.00%	100.00%	100.00%	100.00%
31	QID21444.1	100.00%	100.00%	100.00%	100.00%	100.00%	100.00%
32	QID21428.1	100.00%	100.00%	100.00%	100.00%	100.00%	100.00%
33	APT69981.1	100.00%	100.00%	100.00%	100.00%	100.00%	100.00%
34	AID60235.1	100.00%	100.00%	100.00%	100.00%	100.00%	100.00%
35	AID60229.1	100.00%	100.00%	100.00%	100.00%	100.00%	100.00%
36	AID60216.1	100.00%	100.00%	100.00%	100.00%	100.00%	100.00%
37	WVE62484.1	100.00%	100.00%	100.00%	100.00%	100.00%	100.00%
38	QID21453.1	100.00%	100.00%	100.00%	100.00%	100.00%	100.00%
39	QDF63513.1	100.00%	100.00%	100.00%	100.00%	100.00%	100.00%
40	QCH41415.1	100.00%	100.00%	100.00%	100.00%	100.00%	100.00%
41	APZ88654.1	100.00%	100.00%	100.00%	100.00%	100.00%	100.00%

42	APT69982.1	100.00%	100.00%	100.00%	100.00%	100.00%	100.00%
43	AID60226.1	100.00%	100.00%	100.00%	100.00%	100.00%	100.00%
44	WPD49162.1	100.00%	100.00%	100.00%	100.00%	100.00%	100.00%
45	WPD49161.1	100.00%	100.00%	100.00%	100.00%	100.00%	100.00%
46	QKG86109.1	100.00%	100.00%	100.00%	100.00%	100.00%	100.00%
47	ARX73850.1	100.00%	100.00%	100.00%	100.00%	100.00%	100.00%
48	XXP31295.1	100.00%	100.00%	100.00%	100.00%	100.00%	100.00%
49	AID60239.1	100.00%	100.00%	100.00%	100.00%	100.00%	100.00%
50	AID60221.1	100.00%	100.00%	100.00%	100.00%	100.00%	100.00%
51	YBL38112.1	100.00%	100.00%	100.00%	100.00%	100.00%	100.00%
52	WEM02437.1	100.00%	100.00%	100.00%	100.00%	100.00%	100.00%
53	WEM02435.1	100.00%	100.00%	100.00%	100.00%	100.00%	100.00%
54	QKG86108.1	100.00%	100.00%	100.00%	100.00%	100.00%	100.00%
55	AXU25876.1	100.00%	100.00%	100.00%	100.00%	100.00%	100.00%
56	QDF63514.1	100.00%	100.00%	100.00%	100.00%	100.00%	100.00%
57	APZ88661.1	100.00%	100.00%	100.00%	100.00%	100.00%	100.00%
58	AID60227.1	100.00%	100.00%	100.00%	100.00%	100.00%	100.00%
59	WQA41500.1	100.00%	100.00%	100.00%	100.00%	100.00%	100.00%
60	QID21466.1	100.00%	100.00%	100.00%	100.00%	100.00%	100.00%
61	QID21426.1	100.00%	100.00%	100.00%	100.00%	100.00%	100.00%
62	AID60238.1	100.00%	100.00%	100.00%	100.00%	100.00%	100.00%
63	QID21477.1	100.00%	100.00%	100.00%	100.00%	100.00%	100.00%
64	CAD7708028.1	100.00%	100.00%	100.00%	100.00%	100.00%	100.00%
65	CAD7708025.1	100.00%	100.00%	100.00%	100.00%	100.00%	100.00%
66	AQA99456.1	100.00%	100.00%	100.00%	100.00%	100.00%	100.00%
67	API68248.1	100.00%	100.00%	100.00%	100.00%	100.00%	100.00%
68	YBL38006.1	100.00%	100.00%	100.00%	100.00%	100.00%	100.00%
69	WQA41496.1	100.00%	100.00%	100.00%	100.00%	100.00%	100.00%
70	AQT03225.1	100.00%	100.00%	100.00%	100.00%	100.00%	100.00%
71	AQT03224.1	100.00%	100.00%	100.00%	100.00%	100.00%	100.00%
72	AOP04274.1	100.00%	100.00%	100.00%	100.00%	100.00%	100.00%
73	XOF08393.1	100.00%	100.00%	100.00%	100.00%	100.00%	100.00%
74	AQT03215.1	100.00%	100.00%	100.00%	100.00%	100.00%	100.00%
75	APQ43025.1	100.00%	100.00%	100.00%	100.00%	100.00%	100.00%
76	AXU25881.1	100.00%	100.00%	100.00%	100.00%	100.00%	100.00%
77	AXU25878.1	100.00%	100.00%	100.00%	100.00%	100.00%	100.00%
78	AGP50645.1	100.00%	100.00%	100.00%	100.00%	100.00%	93.33%
79	WEM02444.1	100.00%	100.00%	100.00%	100.00%	100.00%	93.33%
80	WEM02442.1	100.00%	100.00%	100.00%	100.00%	100.00%	100.00%
81	QCD26210.1	100.00%	100.00%	100.00%	100.00%	100.00%	100.00%
82	AXU25882.1	100.00%	100.00%	100.00%	100.00%	100.00%	100.00%
83	WMP24406.3	100.00%	100.00%	100.00%	88.89%	93.33%	100.00%
84	WMP24395.3	100.00%	100.00%	100.00%	88.89%	93.33%	100.00%
85	WEM02443.1	100.00%	100.00%	100.00%	100.00%	100.00%	100.00%
86	QCD26211.1	100.00%	100.00%	100.00%	100.00%	100.00%	100.00%
87	YBL38126.1	100.00%	100.00%	100.00%	88.89%	93.33%	100.00%

88	WMP24408.3	100.00%	100.00%	100.00%	88.89%	93.33%	100.00%
89	WMP24377.2	100.00%	100.00%	100.00%	88.89%	93.33%	100.00%
90	QKG86112.1	100.00%	100.00%	100.00%	100.00%	100.00%	100.00%
91	QCD26212.1	100.00%	100.00%	100.00%	100.00%	100.00%	100.00%
92	QCD26209.1	100.00%	100.00%	100.00%	100.00%	100.00%	100.00%
93	XOF08394.1	100.00%	100.00%	100.00%	88.89%	93.33%	100.00%
94	AXU25880.1	100.00%	100.00%	100.00%	100.00%	100.00%	100.00%
95	AWI66603.1	100.00%	100.00%	100.00%	100.00%	100.00%	100.00%
96	XPZ21238.1	100.00%	100.00%	100.00%	88.89%	93.33%	100.00%
97	WEM02440.1	100.00%	100.00%	100.00%	100.00%	100.00%	100.00%
98	XOF08381.1	100.00%	100.00%	100.00%	88.89%	93.33%	100.00%
99	QCD26213.1	93.75%	100.00%	100.00%	100.00%	100.00%	100.00%
100	YBL38147.1	100.00%	100.00%	100.00%	88.89%	93.33%	100.00%
101	YBL38129.1	100.00%	100.00%	100.00%	88.89%	93.33%	100.00%
102	YBL38060.1	100.00%	100.00%	100.00%	88.89%	93.33%	100.00%
103	YBL38038.1	100.00%	100.00%	100.00%	88.89%	93.33%	100.00%
104	WMP24425.3	100.00%	100.00%	100.00%	88.89%	93.33%	100.00%
105	WMP24387.2	93.75%	100.00%	100.00%	100.00%	100.00%	100.00%
106	WMP24386.2	93.75%	100.00%	100.00%	100.00%	100.00%	100.00%
107	WMP24380.2	100.00%	100.00%	100.00%	88.89%	93.33%	100.00%
108	YBL38142.1	100.00%	100.00%	100.00%	88.89%	93.33%	100.00%
109	YBL38135.1	100.00%	100.00%	100.00%	88.89%	93.33%	100.00%
110	YBL38132.1	100.00%	100.00%	100.00%	88.89%	93.33%	100.00%
111	YBL38130.1	100.00%	100.00%	100.00%	88.89%	93.33%	100.00%
112	YBL38120.1	100.00%	100.00%	100.00%	88.89%	93.33%	100.00%
113	YBL38117.1	100.00%	100.00%	100.00%	88.89%	93.33%	100.00%
114	YBL38113.1	100.00%	100.00%	100.00%	88.89%	93.33%	100.00%
115	YBL38109.1	100.00%	100.00%	100.00%	88.89%	93.33%	100.00%
116	YBL38102.1	100.00%	100.00%	100.00%	88.89%	93.33%	100.00%
117	YBL38099.1	100.00%	100.00%	100.00%	88.89%	93.33%	100.00%
118	YBL38096.1	100.00%	100.00%	100.00%	88.89%	93.33%	100.00%
119	YBL38095.1	100.00%	100.00%	100.00%	88.89%	93.33%	100.00%
120	YBL38094.1	100.00%	100.00%	100.00%	88.89%	93.33%	100.00%
121	YBL38088.1	100.00%	100.00%	100.00%	88.89%	93.33%	100.00%
122	YBL38086.1	100.00%	100.00%	100.00%	88.89%	93.33%	100.00%
123	YBL38082.1	100.00%	100.00%	100.00%	88.89%	93.33%	100.00%
124	YBL38080.1	100.00%	100.00%	100.00%	88.89%	93.33%	100.00%
125	YBL38078.1	100.00%	100.00%	100.00%	88.89%	93.33%	100.00%
126	YBL38077.1	100.00%	100.00%	100.00%	88.89%	93.33%	100.00%
127	YBL38071.1	100.00%	100.00%	100.00%	88.89%	93.33%	100.00%
128	YBL38069.1	100.00%	100.00%	100.00%	88.89%	93.33%	100.00%
129	YBL38061.1	100.00%	100.00%	100.00%	88.89%	93.33%	100.00%
130	YBL38058.1	100.00%	100.00%	100.00%	88.89%	93.33%	100.00%
131	YBL38054.1	100.00%	100.00%	100.00%	88.89%	93.33%	100.00%
132	YBL38050.1	100.00%	100.00%	100.00%	88.89%	93.33%	100.00%
133	YBL38047.1	100.00%	100.00%	100.00%	88.89%	93.33%	100.00%

134	YBL38042.1	100.00%	100.00%	100.00%	88.89%	93.33%	100.00%
135	YBL38032.1	100.00%	100.00%	100.00%	88.89%	93.33%	100.00%
136	YBL38027.1	100.00%	100.00%	100.00%	88.89%	93.33%	100.00%
137	YBL38025.1	100.00%	100.00%	100.00%	88.89%	93.33%	100.00%
138	YBL38024.1	100.00%	100.00%	100.00%	88.89%	93.33%	100.00%
139	YBL38011.1	100.00%	100.00%	100.00%	88.89%	93.33%	100.00%
140	YBL38010.1	100.00%	100.00%	100.00%	88.89%	93.33%	100.00%
141	YBL38008.1	100.00%	100.00%	100.00%	88.89%	93.33%	100.00%
142	XLT96473.1	100.00%	100.00%	100.00%	88.89%	93.33%	100.00%
143	XBA14356.1	100.00%	100.00%	100.00%	88.89%	93.33%	100.00%
144	AXU25883.1	100.00%	100.00%	100.00%	100.00%	100.00%	100.00%
145	AXU25879.1	100.00%	100.00%	100.00%	88.89%	93.33%	100.00%
146	ASV46653.1	100.00%	100.00%	100.00%	88.89%	93.33%	100.00%
147	AAS59401.1	100.00%	100.00%	100.00%	100.00%	100.00%	100.00%
148	YBL38149.1	100.00%	100.00%	100.00%	88.89%	93.33%	100.00%
149	YBL38146.1	100.00%	100.00%	100.00%	88.89%	93.33%	100.00%
150	YBL38144.1	100.00%	100.00%	100.00%	88.89%	93.33%	100.00%
151	YBL38139.1	100.00%	100.00%	100.00%	88.89%	93.33%	100.00%
152	YBL38128.1	100.00%	100.00%	100.00%	88.89%	93.33%	100.00%
153	YBL38127.1	100.00%	100.00%	100.00%	88.89%	93.33%	100.00%
154	YBL38123.1	100.00%	100.00%	100.00%	88.89%	93.33%	100.00%
155	YBL38122.1	100.00%	100.00%	100.00%	88.89%	93.33%	100.00%
156	YBL38121.1	100.00%	100.00%	100.00%	88.89%	93.33%	100.00%
157	YBL38118.1	100.00%	100.00%	100.00%	88.89%	93.33%	100.00%
158	YBL38116.1	100.00%	100.00%	100.00%	88.89%	93.33%	100.00%
159	YBL38114.1	100.00%	100.00%	100.00%	88.89%	93.33%	100.00%
160	YBL38111.1	100.00%	100.00%	100.00%	88.89%	93.33%	100.00%
161	YBL38110.1	100.00%	100.00%	100.00%	88.89%	93.33%	100.00%
162	YBL38108.1	100.00%	100.00%	100.00%	88.89%	93.33%	100.00%
163	YBL38107.1	100.00%	100.00%	100.00%	88.89%	93.33%	100.00%
164	YBL38105.1	100.00%	100.00%	100.00%	88.89%	93.33%	100.00%
165	YBL38104.1	100.00%	100.00%	100.00%	88.89%	93.33%	100.00%
166	YBL38103.1	100.00%	100.00%	100.00%	88.89%	93.33%	100.00%
167	YBL38101.1	100.00%	100.00%	100.00%	88.89%	93.33%	100.00%
168	YBL38090.1	100.00%	100.00%	100.00%	88.89%	93.33%	100.00%
169	YBL38087.1	100.00%	100.00%	100.00%	88.89%	93.33%	100.00%
170	YBL38075.1	100.00%	100.00%	100.00%	88.89%	93.33%	100.00%
171	YBL38074.1	100.00%	100.00%	100.00%	88.89%	93.33%	100.00%
172	YBL38073.1	100.00%	100.00%	100.00%	88.89%	93.33%	100.00%
173	YBL38070.1	100.00%	100.00%	100.00%	88.89%	93.33%	100.00%
174	YBL38068.1	100.00%	100.00%	100.00%	88.89%	93.33%	100.00%
175	YBL38062.1	100.00%	100.00%	100.00%	88.89%	93.33%	100.00%
176	YBL38059.1	100.00%	100.00%	100.00%	88.89%	93.33%	100.00%
177	YBL38057.1	100.00%	100.00%	100.00%	88.89%	93.33%	100.00%
178	YBL38056.1	100.00%	100.00%	100.00%	88.89%	93.33%	100.00%
179	YBL38055.1	100.00%	100.00%	100.00%	88.89%	93.33%	100.00%

180	YBL38052.1	100.00%	100.00%	100.00%	88.89%	93.33%	100.00%
181	YBL38046.1	100.00%	100.00%	100.00%	88.89%	93.33%	100.00%
182	YBL38045.1	100.00%	100.00%	100.00%	88.89%	93.33%	100.00%
183	YBL38039.1	100.00%	93.75%	100.00%	88.89%	93.33%	93.33%
184	YBL38037.1	100.00%	100.00%	100.00%	88.89%	93.33%	100.00%
185	YBL38035.1	100.00%	100.00%	100.00%	88.89%	93.33%	100.00%
186	YBL38029.1	100.00%	100.00%	100.00%	77.78%	53.33%	100.00%
187	YBL38022.1	100.00%	100.00%	100.00%	88.89%	93.33%	100.00%
188	YBL38021.1	100.00%	100.00%	100.00%	88.89%	93.33%	100.00%
189	YBL38020.1	100.00%	100.00%	100.00%	88.89%	93.33%	100.00%
190	YBL38019.1	100.00%	100.00%	100.00%	88.89%	93.33%	100.00%
191	YBL38018.1	100.00%	100.00%	100.00%	88.89%	93.33%	100.00%
192	YBL38013.1	100.00%	100.00%	100.00%	88.89%	93.33%	100.00%
193	YBL38009.1	100.00%	100.00%	100.00%	88.89%	93.33%	100.00%
194	XLT96471.1	100.00%	100.00%	100.00%	88.89%	93.33%	100.00%
195	WMP24392.3	93.75%	100.00%	100.00%	88.89%	93.33%	100.00%
196	WMP24390.2	93.75%	100.00%	100.00%	88.89%	93.33%	100.00%
197	UYD43064.1	100.00%	100.00%	100.00%	88.89%	93.33%	100.00%
198	ASV46652.1	100.00%	100.00%	100.00%	88.89%	93.33%	100.00%
199	AQA99457.1	100.00%	100.00%	100.00%	88.89%	93.33%	100.00%
200	YBL38136.1	100.00%	100.00%	100.00%	88.89%	93.33%	100.00%
