# Supplementary material for: Design and Evaluation of a Multi-Epitope Vaccine Targeting Conserved Envelope and NS5 Proteins of Usutu Virus Using Immunoinformatics
Source: Microorganisms. 2026 Sep 11;14(9):2026. doi: 10.3390/microorganisms14092026 (PMC13609697; doi:10.3390/microorganisms14092026)
Supplement: Supplementary file 1 [file microorganisms-14-02026-s001.zip › Supplementary Material_S4/NS5 Protein Sequence Conservation Analysis.pdf]

Supplementary Table S2. Sequence conservation of selected epitopes from NS5 protein across 200 protein sequences

Sr. No.	Epitopes / Acession No.	FMWLGARFL	NALHFLNSM	VQKLGYILR	WLGARFLEF	KLGEFGKAKGSRAIW
1	APZ88654.1	100%	100%	100%	100%	100%
2	WQA41498.1	100%	100%	100%	100%	100%
3	WPD49163.1	100%	100%	100%	100%	100%
4	WGU19507.1	100%	100%	100%	100%	100%
5	WEM02444.1	100%	100%	100%	100%	100%
6	WEM02438.1	100%	100%	100%	100%	100%
7	AOP04274.1	100%	100%	100%	100%	100%
8	AID60239.1	100%	100%	100%	100%	100%
9	AID60227.1	100%	100%	100%	100%	100%
10	AID60226.1	100%	100%	100%	100%	100%
11	AID60216.1	100%	100%	100%	100%	100%
12	YBL38152.1	100%	100%	100%	100%	100%
13	YBL38141.1	100%	100%	100%	100%	100%
14	YBL38140.1	100%	100%	100%	100%	100%
15	YBL38112.1	100%	100%	100%	100%	100%
16	YBL38012.1	100%	100%	100%	100%	100%
17	XXP31295.1	100%	100%	100%	100%	100%
18	XOF08382.1	100%	100%	100%	100%	100%
19	WVE62484.1	100%	100%	100%	100%	100%
20	WVE62481.1	100%	100%	100%	100%	100%
21	WQA41503.1	100%	100%	100%	100%	100%
22	WQA41500.1	100%	100%	100%	100%	100%
23	WMP24437.3	100%	100%	100%	100%	100%
24	WMP24398.3	100%	100%	100%	100%	100%
25	WMP24395.3	100%	100%	100%	100%	100%
26	WMP24390.2	100%	100%	100%	100%	100%
27	WMP24387.2	100%	100%	100%	100%	100%
28	WMP24385.3	100%	100%	100%	100%	100%
29	WEM02443.1	100%	100%	100%	100%	100%
30	WEM02442.1	100%	100%	100%	100%	100%
31	QCD26212.1	100%	100%	100%	100%	100%
32	AQT03224.1	100%	100%	100%	100%	100%
33	AQA99456.1	100%	100%	100%	100%	100%
34	APZ88661.1	100%	100%	100%	100%	100%
35	APT69982.1	100%	100%	100%	100%	100%
36	APT69981.1	100%	100%	100%	100%	100%
37	APQ43025.1	100%	100%	100%	100%	100%
38	API68248.1	100%	100%	100%	100%	100%
39	AIN76231.1	100%	100%	100%	100%	100%
40	AID60238.1	100%	100%	100%	100%	100%
41	AID60235.1	100%	100%	100%	100%	100%
42	AID60232.1	100%	100%	100%	100%	100%

43	AID60229.1	100%	100%	100%	100%	100%
44	AID60221.1	100%	100%	100%	100%	100%
45	AID60201.1	100%	100%	100%	100%	100%
46	AGP50645.1	100%	100%	100%	100%	100%
47	AAS59401.1	100%	100%	100%	100%	100%
48	YBL38137.1	100%	100%	100%	100%	100%
49	XQH04650.1	100%	100%	100%	100%	100%
50	WVE62480.1	100%	100%	100%	100%	100%
51	WVE62479.1	100%	100%	100%	100%	100%
52	WQA41506.1	100%	100%	100%	100%	100%
53	WQA41497.1	100%	100%	100%	100%	100%
54	WQA41494.1	100%	100%	100%	100%	100%
55	WMP24406.3	100 %	100%	100%	100%	100%
56	WMP24386.2	100%	100%	100%	100%	100%
57	WGU19502.1	100%	100%	100%	100%	100%
58	QKG86112.1	100%	100%	100%	100%	100%
59	QKG86108.1	100%	100%	100%	100%	100%
60	QID21444.1	100%	100%	100%	100%	100%
61	QID21428.1	100%	100%	100%	100%	100%
62	QDF63514.1	100%	100%	100%	100%	100%
63	QCD26211.1	100%	100%	100%	100%	100%
64	CAD7708028.1	100%	100%	100%	100%	100%
65	CAD7708025.1	100%	100%	100%	100%	100%
66	AXU25882.1	100%	100%	100%	100%	100%
67	AQT03225.1	100%	100%	100%	100%	100%
68	YBL38135.1	100%	100%	100%	100%	100%
69	YBL38134.1	100%	100%	100%	100%	100%
70	YBL38132.1	100%	100%	100%	100%	100%
71	YBL38120.1	100%	100%	100%	100%	100%
72	YBL38102.1	100%	100%	100%	100%	100%
73	YBL38093.1	100%	100%	100%	100%	100%
74	YBL38086.1	100%	100%	100%	100%	100%
75	YBL38068.1	100%	100%	100%	100%	100%
76	YBL38065.1	100%	100%	100%	100%	100%
77	YBL38063.1	100%	100%	100%	100%	100%
78	QID21514.1	100%	100%	100%	100%	100%
79	QID21477.1	100%	100%	100%	100%	100%
80	QID21443.1	100%	100%	100%	100%	100%
81	QID21437.1	100%	100%	100%	100%	100%
82	QID21426.1	100%	100%	100%	100%	100%
83	QDF63513.1	100%	100%	100%	100%	100%
84	QCD26213.1	100%	100%	100%	100%	100%
85	QCD26210.1	100%	100%	100%	100%	100%
86	AXU25883.1	100%	100%	100%	100%	100%
87	AXU25881.1	100%	100%	100%	100%	100%
88	AXU25880.1	100%	100%	100%	100%	100%

89	AXU25879.1	100%	100%	100%	100%	100%
90	AXU25876.1	100%	100%	100%	100%	100%
91	AQT03211.1	100%	100%	100%	100%	100%
92	AIW82673.1	100%	100%	100%	100%	100%
93	QID21449.1	100%	100%	100%	100%	100%
94	YBL38153.1	100%	100%	100%	100%	100%
95	YBL38151.1	100%	100%	100%	100%	100%
96	YBL38149.1	100%	100%	100%	100%	100%
97	YBL38148.1	100%	100%	100%	100%	100%
98	YBL38147.1	100%	100%	100%	100%	100%
99	YBL38146.1	100%	100%	100%	100%	100%
100	YBL38144.1	100%	100%	100%	100%	100%
101	YBL38142.1	100%	100%	100%	100%	100%
102	YBL38139.1	100%	100%	100%	100%	100%
103	YBL38138.1	100%	100%	100%	100%	100%
104	YBL38126.1	100%	100%	100%	100%	100%
105	YBL38123.1	100%	100%	100%	100%	100%
106	YBL38122.1	100%	100%	100%	100%	100%
107	YBL38121.1	100%	100%	100%	100%	100%
108	YBL38118.1	100%	100%	100%	100%	100%
109	YBL38116.1	100%	100%	100%	100%	100%
110	YBL38115.1	100%	100%	100%	100%	100%
111	YBL38114.1	100%	100%	100%	100%	100%
112	YBL38113.1	100%	100%	100%	100%	100%
113	YBL38109.1	100%	100%	100%	100%	100%
114	YBL38101.1	100%	100%	100%	100%	100%
115	YBL38096.1	100%	100%	100%	100%	100%
116	YBL38095.1	100%	100%	100%	100%	100%
117	YBL38094.1	100%	100%	100%	100%	100%
118	YBL38090.1	100%	100%	100%	100%	100%
119	YBL38088.1	100%	100%	100%	100%	100%
120	YBL38087.1	100%	100%	100%	100%	100%
121	YBL38084.1	100%	100%	100%	100%	100%
122	YBL38083.1	100%	100%	100%	100%	100%
123	YBL38082.1	100%	100%	100%	100%	100%
124	YBL38080.1	100%	100%	100%	100%	100%
125	YBL38078.1	100%	100%	100%	100%	100%
126	YBL38075.1	100%	100%	100%	100%	100%
127	YBL38074.1	100%	100%	100%	100%	100%
128	YBL38070.1	100%	100%	100%	100%	100%
129	YBL38058.1	100%	100%	100%	100%	100%
130	YBL38057.1	100%	100%	100%	100%	100%
131	YBL38056.1	100%	100%	100%	100%	100%
132	YBL38054.1	100%	100%	100%	100%	100%
133	YBL38052.1	100%	100%	100%	100%	100%
134	YBL38046.1	100%	100%	100%	100%	100%

135	YBL38045.1	100%	100%	100%	100%	100%
136	YBL38044.1	100%	100%	100%	100%	100%
137	YBL38042.1	100%	100%	100%	100%	100%
138	YBL38039.1	100%	100%	100%	100%	100%
139	YBL38036.1	100%	100%	100%	100%	100%
140	YBL38034.1	100%	100%	100%	100%	100%
141	YBL38031.1	100%	100%	100%	100%	100%
142	YBL38030.1	100%	100%	100%	100%	100%
143	YBL38029.1	100%	100%	100%	100%	100%
144	YBL38024.1	100%	100%	100%	100%	100%
145	YBL38022.1	100%	100%	100%	100%	100%
146	YBL38021.1	100%	100%	100%	100%	100%
147	YBL38020.1	100%	100%	100%	100%	100%
148	YBL38019.1	100%	100%	100%	100%	100%
149	YBL38018.1	100%	100%	100%	100%	100%
150	YBL38013.1	100%	100%	100%	100%	100%
151	YBL38010.1	100%	100%	100%	100%	100%
152	YBL38009.1	100%	100%	100%	100%	100%
153	YBL38008.1	100%	100%	100%	100%	100%
154	YBL38005.1	100%	100%	100%	100%	100%
155	XPZ21238.1	100%	100%	100%	100%	100%
156	XOF08391.1	100%	100%	100%	100%	100%
157	XOF08386.1	100%	100%	100%	100%	100%
158	WMP24430.3	100%	100%	100%	100%	100%
159	WMP24425.3	100%	100%	100%	100%	100%
160	WMP24408.3	100%	100%	100%	100%	100%
161	WMP24399.3	100%	100%	100%	100%	100%
162	WMP24397.3	100%	100%	100%	100%	100%
163	WMP24393.2	100%	100%	100%	100%	100%
164	WMP24392.3	100%	100%	100%	100%	100%
165	WMP24378.2	100%	100%	100%	100%	100%
166	WMP24377.2	100%	100%	100%	100%	100%
167	UKC75731.1	100%	100%	100%	100%	100%
168	QKG86110.1	100%	100%	100%	100%	100%
169	QID21520.1	100%	100%	100%	100%	100%
170	QID21518.1	100%	100%	100%	100%	100%
171	QID21501.1	100%	100%	100%	100%	100%
172	QID21498.1	100%	100%	100%	100%	100%
173	QID21459.1	100%	100%	100%	100%	100%
174	QID21452.1	100%	100%	100%	100%	100%
175	QID21442.1	100%	100%	100%	100%	100%
176	QID21422.1	100%	100%	100%	100%	100%
177	AQT03218.1	100%	100%	100%	100%	100%
178	YBL38143.1	100%	100%	100%	100%	100%
179	YBL38117.1	100%	100%	100%	100%	100%
180	YBL38111.1	100%	100%	100%	100%	100%

181	YBL38107.1	100%	100%	100%	100%	100%
182	YBL38106.1	100%	100%	100%	100%	100%
183	YBL38104.1	100%	100%	100%	100%	100%
184	YBL38103.1	100%	100%	100%	100%	100%
185	YBL38077.1	100%	100%	100%	100%	100%
186	YBL38069.1	100%	100%	100%	100%	100%
187	YBL38059.1	100%	100%	100%	100%	100%
188	YBL38050.1	100%	100%	100%	100%	100%
189	YBL38047.1	100%	100%	100%	100%	100%
190	YBL38035.1	100%	100%	100%	100%	100%
191	YBL38027.1	100%	100%	100%	100%	100%
192	YBL38025.1	100%	100%	100%	100%	100%
193	XOF08394.1	100%	100%	100%	100%	100%
194	XBA14356.1	100%	100%	100%	100%	100%
195	WMP24396.2	100%	100%	100%	100%	100%
196	WMP24380.2	100%	100%	100%	100%	100%
197	UYD43070.1	100%	100%	100%	100%	100%
198	UYD43069.1	100%	100%	100%	100%	100%
199	UYD43067.1	100%	100%	100%	100%	100%
200	UYD43064.1	100%	100%	100%	100%	100%
