# Supplementary material for: Design and Evaluation of a Multi-Epitope Vaccine Targeting Conserved Envelope and NS5 Proteins of Usutu Virus Using Immunoinformatics
Source: Microorganisms. 2026 Sep 11;14(9):2026. doi: 10.3390/microorganisms14092026 (PMC13609697; doi:10.3390/microorganisms14092026)
Supplement: Supplementary file 1 [file microorganisms-14-02026-s001.zip › Supplementary Material_S5/Docking Scores and complexes/Vaccine-TLR2 ClusPro scores.pdf]

Vaccine-TLR2 ClusPro scores

Cluster	Members	Representative	Weighted Score
**0**	59	Center	-1181.8
		Lowest Energy	-1326.1
**1**	42	Center	-1180.7
		Lowest Energy	-1280.1
**2**	33	Center	-1168.6
		Lowest Energy	-1313.9
**3**	32	Center	-1273.0
		Lowest Energy	-1491.9
**4**	29	Center	-1216.8
		Lowest Energy	-1472.7
**5**	29	Center	-1196.7
		Lowest Energy	-1434.8
**6**	28	Center	-1117.8
		Lowest Energy	-1475.3
**7**	28	Center	-1244.4
		Lowest Energy	-1376.7
**8**	26	Center	-1168.9
		Lowest Energy	-1202.0
**9**	26	Center	-1156.3
		Lowest Energy	-1286.1
**10**	25	Center	-1285.0
		Lowest Energy	-1385.8
**11**	24	Center	-1431.9
		Lowest Energy	-1431.9
**12**	21	Center	-1112.5
		Lowest Energy	-1393.9
**13**	20	Center	-1151.7
		Lowest Energy	-1296.9
**14**	18	Center	-1131.9
		Lowest Energy	-1259.2
**15**	18	Center	-1344.9

Cluster	Members	Representative	Weighted Score
		Lowest Energy	-1344.9
**16**	14	Center	-1221.4
		Lowest Energy	-1358.7
**17**	14	Center	-1124.6
		Lowest Energy	-1408.1
**18**	14	Center	-1123.0
		Lowest Energy	-1387.1
**19**	14	Center	-1135.9
		Lowest Energy	-1260.2
**20**	13	Center	-1173.9
		Lowest Energy	-1308.5
**21**	13	Center	-1307.2
		Lowest Energy	-1307.2
**22**	13	Center	-1097.2
		Lowest Energy	-1247.9
**23**	12	Center	-1228.4
		Lowest Energy	-1228.4
**24**	12	Center	-1192.1
		Lowest Energy	-1243.9
**25**	12	Center	-1117.4
		Lowest Energy	-1323.3
**26**	11	Center	-1149.4
		Lowest Energy	-1366.8
**27**	11	Center	-1129.1
		Lowest Energy	-1285.3
**28**	10	Center	-1126.7
		Lowest Energy	-1208.4
**29**	10	Center	-1178.6
		Lowest Energy	-1183.3
