# Supplementary material for: Design and Evaluation of a Multi-Epitope Vaccine Targeting Conserved Envelope and NS5 Proteins of Usutu Virus Using Immunoinformatics
Source: Microorganisms. 2026 Sep 11;14(9):2026. doi: 10.3390/microorganisms14092026 (PMC13609697; doi:10.3390/microorganisms14092026)
Supplement: Supplementary file 1 [file microorganisms-14-02026-s001.zip › Supplementary Material_S5/Docking Scores and complexes/Vaccine-TLR4 ClusPro scores.pdf]

Vaccine-TLR4 ClusPro scores

Cluster	Members	Representative	Weighted Score
**0**	30	Center	-1124.8
		Lowest Energy	-1230.2
**1**	25	Center	-1152.5
		Lowest Energy	-1278.2
**2**	25	Center	-1152.2
		Lowest Energy	-1454.3
**3**	24	Center	-1379.5
		Lowest Energy	-1379.5
**4**	23	Center	-1138.7
		Lowest Energy	-1270.2
**5**	22	Center	-1363.7
		Lowest Energy	-1363.7
**6**	22	Center	-1363.5
		Lowest Energy	-1409.0
**7**	20	Center	-1547.5
		Lowest Energy	-1547.5
**8**	20	Center	-1237.4
		Lowest Energy	-1313.2
**9**	19	Center	-1215.9
		Lowest Energy	-1378.7
**10**	19	Center	-1497.2
		Lowest Energy	-1497.2
**11**	18	Center	-1124.9
		Lowest Energy	-1363.2
**12**	18	Center	-1194.2
		Lowest Energy	-1284.9
**13**	16	Center	-1185.5
		Lowest Energy	-1372.7
**14**	15	Center	-1163.3

Cluster	Members	Representative	Weighted Score
		Lowest Energy	-1204.0
**15**	15	Center	-1227.9
		Lowest Energy	-1227.9
**16**	14	Center	-1120.1
		Lowest Energy	-1221.2
**17**	14	Center	-1191.9
		Lowest Energy	-1236.4
**18**	14	Center	-1290.0
		Lowest Energy	-1290.0
**19**	13	Center	-1352.7
		Lowest Energy	-1352.7
**20**	13	Center	-1150.8
		Lowest Energy	-1285.5
**21**	12	Center	-1232.7
		Lowest Energy	-1308.7
**22**	12	Center	-1279.2
		Lowest Energy	-1279.2
**23**	11	Center	-1206.3
		Lowest Energy	-1265.2
**24**	11	Center	-1293.5
		Lowest Energy	-1293.5
**25**	11	Center	-1231.0
		Lowest Energy	-1232.0
**26**	11	Center	-1221.9
		Lowest Energy	-1335.6
**27**	10	Center	-1249.2
		Lowest Energy	-1255.2
**28**	10	Center	-1209.4
		Lowest Energy	-1220.6
**29**	10	Center	-1115.0

Cluster	Members	Representative	Weighted Score
		Lowest Energy	-1217.2
