# Supplementary material for: Design and Evaluation of a Multi-Epitope Vaccine Targeting Conserved Envelope and NS5 Proteins of Usutu Virus Using Immunoinformatics
Source: Microorganisms. 2026 Sep 11;14(9):2026. doi: 10.3390/microorganisms14092026 (PMC13609697; doi:10.3390/microorganisms14092026)
Supplement: Supplementary file 1 [file microorganisms-14-02026-s001.zip › Supplementary Material_S5/Predicted vaccine 3D model/Galaxy refine results.pdf]

Model	GDT-HA	RMSD	MolProbity	Clash score	Poor rotamers	Rama favored
Initial	1.0000	0.000	2.394	7.6	2.8	86.1
MODEL 1	0.9271	0.530	1.081	2.9	0.0	99.2
MODEL 2	0.9250	0.535	1.050	2.6	0.0	99.2
MODEL 3	0.9354	0.485	1.081	2.9	0.6	98.3
MODEL 4	0.9271	0.540	1.050	2.6	0.6	99.2
MODEL 5	0.9312	0.527	1.108	3.2	0.6	99.2
