# Supplementary material for: Design and Evaluation of a Multi-Epitope Vaccine Targeting Conserved Envelope and NS5 Proteins of Usutu Virus Using Immunoinformatics
Source: Microorganisms. 2026 Sep 11;14(9):2026. doi: 10.3390/microorganisms14092026 (PMC13609697; doi:10.3390/microorganisms14092026)
Supplement: Supplementary file 1 [file microorganisms-14-02026-s001.zip › Supplementary Material_S5/Vaccine construct_Amino acid sequence.pdf]

GIINTLQKYYCRVRGGRCAVLSCLPKEEQIGKCSTRGRKCCRRKKEAAAKALPWTSPASSNWRN
REKKYIVVGRGDKQINHHWHAAYWLGARFLEFAAYIPISIVASLAAYFMWLGARFLAAYGLMG
ALLLWAAYNALHFLNSMAAYVQKLGYILRGPGPGKIPISIVASLSDLTPGPGPGPPFGDSYIVVGR
GDKGPGPGKLGEFGKAKGSRAIWGPGPGAKFVAAWTLKAAAHHHHHH
